# Treatment-Resistant Schizophrenia, Clozapine Resistance, Genetic Associations, and Implications for Precision Psychiatry: A Scoping Review

**DOI:** 10.3390/genes14030689

**Published:** 2023-03-10

**Authors:** Jiangbo Ying, Qian Hui Chew, Roger S. McIntyre, Kang Sim

**Affiliations:** 1 East Region, Institute of Mental Health, Singapore 539747, Singapore; 2 Research Division, Institute of Mental Health, Singapore 539747, Singapore; 3 Department of PsychiSatry, University of Toronto, Toronto, ON M5R 0A3, Canada; 4 Brain and Cognition Discovery Foundation Toronto, Toronto, ON M4W 3W4, Canada; 5 West Region, Institute of Mental Health, Singapore 539747, Singapore

**Keywords:** treatment-resistant schizophrenia, clozapine, precision psychiatry

## Abstract

Treatment-resistant schizophrenia (TRS) is often associated with severe burden of disease, poor quality of life and functional impairment. Clozapine is the gold standard for the treatment of TRS, although it is also known to cause significant side effects in some patients. In view of the burgeoning interest in the role of genetic factors in precision psychiatry, we conducted a scoping review to narratively summarize the current genetic factors associated with TRS, clozapine resistance and side effects to clozapine treatment. We searched PubMed from inception to December 2022 and included 104 relevant studies in this review. Extant evidence comprised associations between TRS and clozapine resistance with genetic factors related to mainly dopaminergic and serotoninergic neurotransmitter systems, specifically, TRS and rs4680, rs4818 within COMT, and rs1799978 within DRD2; clozapine resistance and DRD3 polymorphisms, CYP1A2 polymorphisms; weight gain with LEP and SNAP-25 genes; and agranulocytosis risk with HLA-related polymorphisms. Future studies, including replication in larger multi-site samples, are still needed to elucidate putative risk genes and the interactions between different genes and their correlations with relevant clinical factors such as psychopathology, psychosocial functioning, cognition and progressive changes with treatment over time in TRS and clozapine resistance.

## 1. Introduction

Schizophrenia is a debilitating major mental illness, and it affects about 1% of the population [1]. Prevalent cases of schizophrenia globally have been reported to increase from 13.1 million in 1990 to 20.9 million in 2016, contributing almost 13.4 million years of life lived with disability to the global disease burden [2]. The economic burden of schizophrenia in the United States was estimated to be more than USD 60 billion annually [3]. Unfortunately, not all patients with schizophrenia respond adequately to treatment. Although the mainstay of treatment for schizophrenia is antipsychotic medication, approximately one third of patients have a limited response to antipsychotic medication treatment and are assessed to have treatment-resistant schizophrenia (TRS) [4]. Patients with TRS, compared with schizophrenia in remission, are reported to have an increased rate of suicidal ideation [5], impaired cognitive functioning [6], greater medical comorbidities [7], lower quality of life and higher cost of treatment [5]. Several hypotheses have been proposed for the underlying neurobiology of TRS [8]. One hypothesis suggests that TRS is a result of dopamine supersensitivity as a result of a continuous blockade of dopamine receptors by antipsychotic medications [9,10]. Another hypothesis states that TRS is a result of glutamate dysregulation, which in turn stimulates the activity of dopaminergic projections from the midbrain to the striatum, giving rise to positive symptoms of schizophrenia [11,12].

The criteria to assess for TRS have been continuously updated since 1966 [13] and have been mentioned in several practice guidelines, including those from the American Psychiatric Association [14], The Royal Australian and New Zealand College of Psychiatrists [15], and the British Association for Psychopharmacology [16]. In order to establish consensus criteria to standardize the definition of TRS, the Treatment Response and Resistance in Psychosis (TRRIP) working group was formed, and it proposed that the TRS criteria should include the following: (1) current symptoms of at least moderate severity; (2) treatment with at least two different antipsychotic medications; (3) treatment duration of at least 6 weeks with a total daily dose equivalent of at least 600 mg of chlorpromazine; and (4) medication adherence of at least 80% of the prescribed doses taken [17].

Of note, clozapine is considered as a gold standard for the treatment of TRS and is also often considered as a proxy indicator of treatment resistance [18]. Historically, in 1988, Kane and colleagues first demonstrated the efficacy of clozapine in treating TRS [19]. Subsequently, more studies have reported that clozapine exhibits superiority over other antipsychotics for TRS [20,21]. A meta-analysis included 2530 randomly assigned participants in 30 clinical trials and found that clozapine was more effective than conventional neuroleptics in reducing symptoms in patients with TRS [20]. Another meta-analysis involved 1916 independent patients in 12 controlled studies and confirmed that clozapine was superior in controlling psychotic symptoms in TRS [21]. However, clozapine has significant side effects, some of which are severe and life-threatening. Common side effects of clozapine include tachycardia, metabolic syndrome, hyper-salivation and constipation, while severe side effects (with relative lethality (RL) calculated as fatal outcomes out of reported cases) include pneumonia (RL 30%), myocarditis (RL 12%), arrhythmia (RL 5%), seizure (RL 5%) and agranulocytosis (RL 2%) [22,23,24]. There are several factors that can modulate the clozapine effect, including genetic factors and drug–drug interactions. To reduce the risk of clozapine-related side effects, a recent international guideline suggests considering three parameters: (1) a DNA ancestry group, (2) a sex-smoking subgroup, and (3) the presence or absence of clozapine poor metabolizer status [24].

To better understand the underlying biology of TRS and optimize its treatment, there is increasing interest in the genetic factors associated with TRS and clozapine treatment. For example, a recent genetic association study found that an interaction between the dopaminergic and γ-aminobutyric acid (*GABA*) signal intensities could differentiate patients with and without TRS [25]. Another recent study reported that the Glutamate Decarboxylase 1 (***GAD1***) gene and the GABA Type B Receptor 2 (*GABBR2*) gene were associated with TRS [26]. Other than genes related to TRS, genes associated with clozapine treatment are explored in other studies as well. One study focused on the variability in cytochrome P450 (***CYP***) enzyme genes and found that *CYP2C19* variants were associated with clozapine response [27]. Another study assessed the genes related to clozapine side effects and demonstrated that polymorphism in the α 2A adrenergic receptor gene was associated with clozapine-induced sialorrhea [28].

The aim of the current scoping review is to provide a comprehensive and up-to-date overview of the genes associated with three main areas, namely (1) TRS, (2) clozapine resistance, and (3) clozapine-related side effects. In the interest of exploring the clinical effects of clozapine, this review will concentrate on the pharmacodynamic genes that are pertinent to clozapine treatment. A better understanding of the underlying genetic underpinnings can potentially help to uncover the complex biology of TRS, including clozapine resistance and side effects, with the hope of better optimized treatments of TRS.

## 2. Materials and Methods

The literature search was performed in the PubMed database by including the keyword “schizophrenia” in the title of the article, linked with a combination of the following keywords: “resistant”, “refractory”, “response”, “treatment”, “clozapine” and “gene” in the title or abstract. We included papers published from database inception to December 2022, removing any duplicates prior to screening. In accordance with the inclusion criteria and exclusion criteria, two authors (JY and QHC) independently screened the retrieved literature. In the case of a disagreement, both authors would discuss with the rest of the team until a consensus was reached. Selection criteria were as follows: (1) original papers published in English, (2) definition of TRS was provided by the study, and/or it was explicitly stated that participants recruited were treatment-resistant or on clozapine, (3) genes related to TRS or clozapine were analyzed, (4) most, if not all, of the participants in the primary group of interest had TRS or were on clozapine, (5) studies on human participants. Exclusion criteria were (1) uncertainty about whether participants were treatment-resistant or on clozapine, (2) studies on animals, (3) there was no main gene(s) of interest investigated in relation to TRS or clozapine resistance, (4) the paper was a meta-analysis or systematic review, (5) sample size smaller than five. For each included study, we extracted variables that included the characteristics of subjects, the definition of TRS, and the significant genetic associations.

## 3. Results

**Figure 1:**
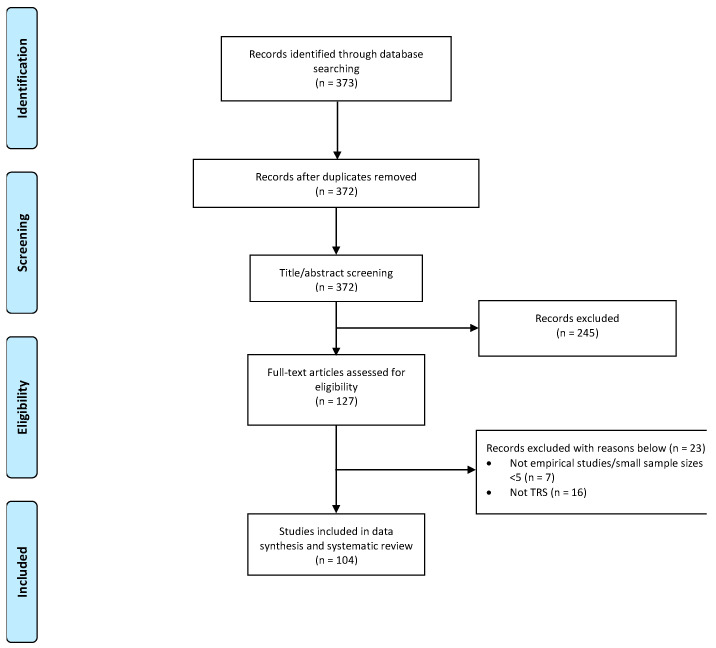
PRISMA chart of studies included in this review.

Overall, 104 studies were included in this review (see Figure 1). The studies were conducted in Asia (*n*= 38, 36.5%), the United States (*n* = 25, 24.0%), Europe (*n* = 23, 22.1%), South America (*n* = 9, 8.65%), both Europe and Asia (*n* = 5, 4.81%), Australia (*n* = 3, 2.88%), and Africa (*n* = 1, 0.96%). The sample size varied from less than 10 to a few thousand, with an average age of 38.8 ± 5.21 years for patients with TRS examined. The proportion of males with TRS across all studies was 66.1%, and the average duration of illness was 16.3 ± 4.54 years.

### 3.1. Risk Genes/Polymorphisms for Treatment-Resistant Schizophrenia 

The most common definition of TRS reported in the included studies consisted of three major elements. First, there is no sufficient improvement in symptoms, or a persistence of moderate-to-severe symptoms, as assessed with the Positive and Negative Syndrome Scale (PANSS), Brief Psychotic Rating Scale (BPRS), and Global Assessment of Functioning (GAF). Second, sufficient doses of two or more trials of antipsychotics (chlorpromazine-equivalent dose of 600 mg or higher) had been used prior to the diagnosis of TRS. Some studies specified that the antipsychotics should be from at least two different biochemical classes. Third, the antipsychotics should have been trialed for a sufficient duration, often defined as a period of at least 4 to 6 weeks. The genes and polymorphisms that are associated with an increased risk of TRS are listed in Table 1. 

#### 3.1.1. Dopaminergic System—Genes Related to TRS

Catechol-O-methyltransferase (*COMT*)

*COMT* plays a key role in the regulation of dopamine activity in the prefrontal cortex and could indirectly affect dopamine levels in the striatum, leading to treatment resistance [55]. Two main single-nucleotide polymorphisms (SNPs) in *COMT* (*rs4680* and *rs4818*) were associated with an increased risk of TRS (See Table 1). *COMT rs4680 met* allele carriers who were male [29], as well as females with the *A/A* genotype [30], were at increased risk. Females with the *COMT rs4818 C/C* genotype were similarly vulnerable [30]. A haplotype of *rs4818-rs4680* non *G-G/G-G* was associated with TRS in females only [30].

Dopamine Receptor D2 (*DRD2*), Dopa Decarboxylase (*DDC*)

The *DRD2* gene codes for the D2 dopamine receptor, which is the primary site of the therapeutic action of antipsychotics [56] and has been implicated in the pathophysiology of schizophrenia [57]. *DRD2 rs1799978* is associated with an increased risk of TRS [32] in *G* allele carriers [33]. *DDC* is an enzyme required for dopamine synthesis, and aberrant dopamine synthesis is hypothesized to underlie symptoms related to schizophrenia [58]. *DDC rs10499696 A/A* genotype carriers were also more susceptible to TRS [32]. *GRB10*, a neighboring gene of *DDC,* was also associated with TRS, with a higher proportion of *rs2237457 T/T* genotype carriers found in the TRS group [42].

Combination of dopaminergic genes

A combination of the *COMT rs4680 met/met* genotype and the *DRD3 rs6280 Ser/Gly* genotype was also predictive of TRS [33].

#### 3.1.2. Serotonergic System—Genes Related to TRS

5-Hydroxytryptamine Receptor 2A (*HTR2A*), 5-Hydroxytryptamine Receptor 2C (*HTR2C*)

Serotonin or 5-hydroxytryptamine (*5-HT*) also plays an important role in schizophrenia. *HTR2C* receptors are able to inhibit dopamine release in the limbic brain areas, and this has been hypothesized to cause psychotic symptoms and affect the efficacy of antipsychotics [59]. Similarly, *HTR2A* receptors showed a reduction in binding in schizophrenia patients and has been implicated in the pathophysiology of schizophrenia [60]. Studies included in our review only reported significant findings for these two genes in male patients. The *HTR2A 2/2* genotype [34], as well as the *HTR2C rs6318 Ser* non-carriers and *HTR2C rs3813929-rs6318* non *C-Ser* haplotype carriers [35] were at increased risk for TRS.

Serotonin Transporter Gene-linked Polymorphic Region (*SERT-PR*)

Given serotonin’s importance in the pathophysiology of schizophrenia, there has been interest in investigating regional changes in *5-HT* sites, such as the *SERT* promoter region. The *SS* genotype of *SERT-PR* was predictive of TRS in a study by Bilic et al. [31].

Combination of dopamine and serotonergic genes

In patients with Dopamine Transporter Gene (SLC6A3) *10/10* or *10/12* genotype, those who had the *SERT-in2 ls* or *ss* genotype were more susceptible to TRS [31]. In patients with the *SERT-in2 ll* genotype, the *SLC6A3 9/10*, *9/11*, *9/9*, or *6/6* genotype was also associated with TRS [31].

#### 3.1.3. GABA/Glutamatergic System—Genes Related to TRS

Glutamate decarboxylase *1* (*GAD1*), γ-Aminobutyric Acid Type B Receptor Subunit 2 (*GABBR2*)

The compound γ aminobutyric acid (*GABA*) is implicated in the pathophysiology of schizophrenia, affecting three main areas relating to learning, memory, and executive functions [61,62]. *GAD1*, the rate-limiting enzyme of *GABA*, is posited to contribute to *GABA* dysfunction in the brain of schizophrenia patients [63]. One study has found showing that *GAD1 rs3749034* and *GABBR2 rs10985765* are related to TRS as well [26].

Glutamate Metabotropic Receptor 3 (*GRM3*)

*GRM3* codes for the *mGluR3* protein, which is needed for optimal glutamate signaling in the brain [64], and it has been associated with response to antipsychotic treatment [65]. The *GRM3 rs1989796 TT* genotype, as well as the *rs1476455 CC* genotype, were associated with TRS [36].

Combination of GABA/Glutamatergic system and dopaminergic system

Kogure and colleagues [25] reported that *COMT rs4680/GAD1 rs3749034 met(+)/T(𢄡)* carriers were more likely to be found in the TRS group.

#### 3.1.4. Other Gene Variants—Genes Related to TRS

Endocannabinoid

The endocannabinoid system is hypothesized to contribute to psychotic symptoms, and the Cannabinoid Receptor 1 (*CNR1*) gene, which codes for endocannabinoid receptors, has been investigated for its possible links to schizophrenia [66]. There was an increase in the expression of *CNR1* found amongst TRS patients, and the *rs806368 T/T* and *T/T* genotype carriers as well as *rs1049353 G/G* genotype carriers [32] had an increased risk of being diagnosed with TRS.

Oxytocin (*OXT*)

Abnormalities in the dopaminergic and oxytocinergic reward system signaling in the amygdala could underlie the social deficits often witnessed in schizophrenia [67]. This ranges from the negative symptoms of withdrawal and isolation to the positive symptoms of suspicion and paranoia [67]. The *OXT rs2740210 C* allele and the *OXTR rs2228485 A* allele as well as the *A/A* genotype were associated with TRS [32].

Brain Derived Neurotrophic Factor (*BDNF*)

Neurodevelopmental models have proposed that synaptic function is negatively affected when *BDNF* concentrations are reduced, altering neurotransmission and giving rise to symptoms seen in schizophrenia [68,69]. Badrlou and colleagues [38] found higher expression levels of *BDNF* as well as the *BDNF*-associated lncRNA *PKNY* in TRS patients. Zhang and colleagues [39] reported an increased incidence of TRS in minor allele carriers of multiple *BDNF* SNPs.

Cytochrome P450s (*CYP*)

*CYP*-related genes code for drug-metabolizing enzymes, such as those involved in metabolizing antipsychotics. TRS was associated with higher mRNA transcript levels of *CYP2A6* and a reduced mRNA expression of *CYP2D6* and *CYP3A4* [40]. Martínez-Magaña and colleagues [41] also reported a loss-of-function variant carrier amongst the TRS group.

MicroRNAs (miRNA) and associated biogenesis machinery

*DICER1* is a main component of the miRNA biogenesis machinery [70] and was upregulated in TRS patients [37]. Expression levels of multiple miRNAs were also elevated in TRS patients, including *miR-181b-5p*, *miR-195-5p*, *miR-301a-3p* [46], *hsa-miR-218-5p*, *hsa-miR-1262* [47], and *hsa-miR-675-3p* [48].

Inflammation and oxidative stress

Oxidative stress has been associated with the pathophysiology of schizophrenia [71], and Glutathione S-transferase (*GST*) is involved in detoxification, thereby protecting cells and tissues from oxidative stress damage. Among the *GSTs*, double-null genotype carriers of *GSTT1* and *GSTM1* were found to have increased risk of TRS [43]. Tumor necrosis factor-α (*TNF*-α) is a proinflammatory cytokine produced by both neurons and glial cells and may be related to psychiatric symptoms [72]. Aytac and colleagues [53] reported a significant relationship between TRS and the *TNF*-α-*238 GG* genotype.

Transcripts within the *NRG*–*ErbB* signaling pathway

Neuregulin-1 (*NRG1*) is involved in various neurodevelopmental processes, including cell migration, synaptic formation, plasticity, and myelination [73]. *NRG1* regulates both excitatory and inhibitory synaptic transmission in the adult brain, and abnormal neurotransmission and/or synaptic plasticity have been reported in both glutamatergic and GABAergic pathways in the brain of schizophrenia patients (e.g., [74]). In our included studies, *NRG1 rs7834206* was found to be associated with TRS [49]. AKT serine/threonine kinase 1 (*AKT1*) acts downstream of the dopamine D2 receptor and has also been associated with schizophrenia in several studies [75,76,77]. It was upregulated in TRS patients in a study by Moretti and colleagues [37]. *P70S6K*, a protein kinase linked to protein synthesis, cell growth, and cell proliferation, was elevated in TRS patients [50].

Genes involved in synaptic functioning

One of the lines of evidence supporting the neurodevelopmental hypothesis of schizophrenia emerges from the finding of abnormal expression of genes involved in neuronal and glial migration, cell proliferation, and synaptogenesis (e.g., [78]). Altered reelin expression may result in the impairment of neuronal connectivity and synaptic plasticity, leading to the cognitive deficits observed in schizophrenia [79]. The study by Goldberger and colleagues [51] provides further support for the involvement of reelin in schizophrenia, with (*CGG*)_*10*_ alleles and genotypes in particular being associated with TRS. Another gene involved in synaptic functioning, SNAP25 synaptosome-associated protein 25 (*SNAP-25*), was found to be related to TRS. The *MnlI T/G* or *G/G* genotype, as well as the *TaiI T/T* or *T/C* genotypes, were associated with an increased risk of TRS [52]. Neurexin 1 (*NRXN1*), which has a fundamental role in synaptogenesis and synaptic maintenance, had a lower methylation rate in TRS patients, as compared to HCs [44].

Ubiquitin-related genes

Ubiquitin fusion degradation protein 1 (*UFD1*) is involved in protein degradation and was found to have increased expression amongst TRS patients [37]. Another ubiquitin conjugating enzyme (Ubiquitin Conjugating Enzyme E2 K; *UBE2K*) was also associated with schizophrenia, and this association is hypothesized to be driven by elevated levels of ubiquitinated proteins [80]. Higher mRNA levels of *UBE2K* have also been found in TRS patients, providing evidence for the involvement of this gene [54].

### 3.2. Risk Genes/Polymorphisms for Clozapine Resistance

Table 2 shows the genes and polymorphisms associated with increased risk for clozapine resistance. Clozapine is often prescribed to patients who have demonstrated inadequate response to at least two antipsychotics. 

The criterion for determining clozapine response/resistance was more varied among the included studies as compared to that of treatment resistance. Most studies employed change scores of rating scales (such as BPRS, PANSS, GAS) from pre-treatment baseline [52,84,101,107,108,109,110,111,112,113,114] or a reduction of more than or equal to 20% in scores, which is often used as an indication of clozapine response [35,81,85,88,96,102,103,106,115,116,117,118,119]. There were also studies that necessitated a greater reduction in scores before considering the patient to be clozapine responsive [83,87,92,104]. A minimum treatment period ranging from 6 to 8 weeks [101,107], 10 weeks [117], and 6 months [81,83,85,103,106] was part of the criteria used by most studies.

#### 3.2.1. Dopaminergic System—Genes Related to Clozapine Resistance

*DRD2*, *DRD3*, *DRD4*

Similar to that of treatment resistance, dopamine receptor genes (*DRD2, DRD3, DRD4*) were found to be related to clozapine resistance (See Table 2). Specifically, *DRD2 rs1799978 T* allele carriers [32], *rs2514218 G/G* genotype carriers [81], *DRD3 Ser 9* allele carriers and *Ser9/Ser9* genotype carriers [83], as well as *1-1* genotype carriers [84] had an increased risk of developing clozapine resistance. As for *DRD4*, most of the risk-gene polymorphisms found to be significantly related to clozapine resistance were specific to either African-Americans or Caucasian patients [85].

Solute Carrier Family 6 Member 3 (*SLC6A3*)

Previous studies suggest that the human dopamine transporter (*SLC6A3*) plays an essential role in the accumulation of extracellular dopamine through which dopamine neurotransmission is controlled, and it is also the main action site of psychostimulants [120,121,122]. Psychotic disturbances have been linked to the aberrant release of dopamine and neurotransmission, which possibly implicates *SLC6A3* in schizophrenia. The *rs2975226-71A* allele, as well as the *rs2652511-844C* × *rs2975226-71A* × *rs2963238-1491C* haplotype, was associated with an increased risk of poor response to clozapine [87].

COMT and combinations with other dopaminergic genes

*COMT rs4680 met* allele carriers were more likely to be clozapine resistant [33]. *COMT val/val* genotype carriers who also had *DRD4 120/120* or *120/140* genotypes had an increased risk of developing clozapine resistance [86].

#### 3.2.2. Serotonergic System—Genes Related to Clozapine Resistance

*HTR2A*, *HTR2C*, *HTR3A*, *HTR3B*

*HTR2A tyr452* allele carriers [88], *HTR3A rs1062613 C* allele or *T/T* genotype carriers [89,90], *rs2276392 A* allele carriers [89], as well as *HTR3B-100_-102delAAG(del)* minor allele carriers [91] were at higher risk of developing clozapine resistance. *HTR2C rs6318 Ser* non-carriers were also more likely to be clozapine resistant, although this effect was significant only among males [35].

Serotonin-Transporter-Linked Promoter Region (SLC6A4/HTTLPR)

The *SLC6A4 rs25531 S*′ allele and the *S*′/*S*′ or *S*′/*L*′ genotypes were associated with clozapine resistance [92].

#### 3.2.3. GABA/Glutamatergic Systems—Genes Associated with Clozapine Resistance

*GAD67* mRNA is an enzyme which has a key role in the production and release of brain *GABA* [123]. Higher mRNA levels of *GAD67*, *GAD25*, and *GAD1* were detected in patients with clozapine resistance [93].

#### 3.2.4. Other Genes Associated with Clozapine Resistance

Endocannabinoid

The *CNR1 rs8006379 C* allele as well as the *rs1043953 A* allele carriers were at higher risk of developing clozapine resistance [32].

*BDNF*

A higher expression of *BDNF* [95] and the *Val*/*Val* genotype [96] were associated with clozapine resistance in two of the included studies.

*CYP*

*CYP1A2* and *CYP2C19* activity scores were elevated and reduced in patients with clozapine resistance, respectively [100]. In addition, the CYP1A2*1F AA and *AC* genotypes [98], as well as the CYP2C19 *1/*17 genotype and *2 allele carriers [27] were at increased risk of developing clozapine resistance. The *CYP1A2-163A* allele was associated with clozapine resistance in smokers only [99].

Drug transporters encoded by the human ATP-binding cassette (*ABC*) gene family are hypothesized to affect the pharmacokinetics and response to clozapine [124]. Breast Cancer Resistance Protein (*BCRP*) encoded by the *ABCG2* gene may be inhibited by clozapine and affect its plasma concentrations [125]. The *ABCG2 421 C/C* genotype was associated with clozapine resistance in a study by Akamine and colleagues [94].

cAMP-Response Element Binding Protein (*CREB*)

The *CREB* binding protein is a co-activator of the *CREB1* gene, which has been associated with response to treatment (in terms of cognitive improvement) in schizophrenia patients [126]. The CpG site *cg05151055* of *CREBBP* showed decreased methylation in patients with clozapine resistance [97].

Mitochondrial uncoupling protein 4 (*SLC25A27*/*UCP4*) regulates the production of reactive oxygen species [127] as well as cellular calcium homeostasis [128] and has a neuroprotective function. Oxidative stress and aberrant calcium signaling affect mitochondrial function and consequently neuronal plasticity and neurotransmission, thereby increasing the risk of schizophrenia [129,130]. Non-carriers of the *CCAC* haplotype of *rs3757241* × *rs10807344* × *rs9395206* × *rs2270450* were more likely to demonstrate poor response to clozapine [107].

##### Genes That Are Involved in Cellular Interactions and Responses

Contactin-associated protein-like 2 (*CNTNAP2*) encodes for a group of transmembrane proteins that control cell–cell interactions in the nervous system [131]. The internal and overlapping deletions in the *CNTNAP2* gene were associated with an increased risk of schizophrenia [132]. Higher mRNA levels of *CNTNAP2* were detected in patients with a poor clozapine response [93].

Guanine nucleotide binding proteins (*G*-proteins) regulate cellular responses. The abnormal expression and function of these proteins [133] and their subunits have been associated with various mental health conditions [134] as well as response to treatment [135]. In our included studies, the *G*-protein β subunit 3 (*GNB3*) *825 T/T* genotype was also associated with a poorer response to clozapine [104].

##### Genes That Affect Neuronal Development

Disrupted in Schizophrenia 1 (*DISC1*) is posited to be involved in synaptic pruning [136], and an over-pruning of cortical synapses during critical neurodevelopmental periods is hypothesized to contribute to the development of schizophrenia [137,138]. The *DISC1 rs3738401* minor allele *A* carriers, *A/A* or *A/G* genotype carriers, as well as *rs6675281 T* allele non-carriers had a poorer response to clozapine [101].

There is evidence to suggest that Dystrobrevin-binding Protein 1 (*DTNBP1*) may be involved in regulating neuronal growth and neurotransmission, contributing to the pathogenesis of schizophrenia as a result [139]. The association between *DTNBP1* and clozapine response was only found in the African-American population for our included studies, with the *rs742105 C* allele carriers and the genotype *C/C* carriers demonstrating poorer responses [102].

Evidence indicates that Glial cell line-derived Neurotrophic Factor (*GDNF*) plays a role in mammalian neuronal development [140], and *GDNF* family receptor α-1 (*GFRA*) proteins act as co-receptors to allow *GDNF* proteins to bind to the receptors [103], suggesting that *GFRA* may be involved in the pathophysiology of schizophrenia. The *GFRA2 1-1-2 SNP27-SNP34-SNP37* haplotype non-carriers showed a poorer response to clozapine in the study by Souza and colleagues [103].

Inositol Monophosphatase 2 (*IMPA2*) is hypothesized to be involved in the phospholipase C signalling pathway, which mediates the action of several neurotransmitters and hormones [141] and which may underlie schizophrenia. Patients with clozapine resistance had higher mRNA levels of *IMPA2* in the study by Sershen and colleagues [93].

A lack of Potassium Inwardly Rectifying Channel Subfamily J Member 10 (*KCNJ10*) in mice has previously been associated with myelin compaction failure and axonal degeneration [142]. In line with this finding, a lower expression of *KCNJ10* was also found in patients with a poorer clozapine response [95].

Ligand Of Numb-Protein X 1 (*LNX1*) is responsible for the proteasomal degradation of *NUMB* protein, which is an important regulator of neurogenesis and neuronal differentiation [143]. *LNX1* expression was higher in patients with a poorer response to clozapine [95].

Nuclear factor 1 B-type (*NFIB*) is necessary for normal cortical formation and development [144,145]. This gene was implicated in clozapine response as well, with the *rs28379954 C* allele associated with a poorer response [105].

*NRXN1* encodes the neurexin-1α protein, the lack of which results in a loss of synaptic strength in the excitatory synapses of the hippocampus [146,147]. The *rs1045881 T* allele was associated with a poorer clozapine response [106].

Serpin Family A Member 5 (*SERPINA5*) encodes a protein that plays a role in synaptic plasticity and memory formation [148]. Lower expression levels of *SERPINA5* were associated with a poorer response to clozapine [95].

Tet Methylcytosine Dioxygenase 1 (*TET1*) appears to be involved in catalyzing 5hmC formation in oligodendrocytes [149] and is crucial for myelin formation and repair [63,150]. Lower mRNA levels of *TET1* were associated with poorer clozapine response in patients [93].

### 3.3. Genes/Polymorphisms Associated with Clozapine Side Effects 

Despite the efficacy of clozapine for treatment-resistant schizophrenia, there are significant side effects associated with its use, some of which are severe and life-threatening [22,23,24]. Table 3 shows the common genes and polymorphisms associated with clozapine side effects in patients.

There were several common side effects reported by the included studies, with the two major categories being metabolic side effects and blood disorders. Low high-density lipoprotein was found in patients with the *DRD2 141 Ins C* allele homozygous genotype [151]. Weight gain was associated with the Leptin (*LEP*)-*2548A/G* polymorphism in two studies, with the *A/A* genotype in Kang et al. [157], and the *G/A* or *G/G* genotypes in Zhang et al. [122]. The *SNAP-25 MnlI T/T* genotype as well as the TaiI C/C genotype were associated with weight gain in another separate study [52]. Higher blood pressure, which is also part of metabolic syndrome, was found in *SH2B1* minor allele carriers [158]. Metabolic syndrome as a whole was also linked to the presence of the Sterol Regulatory Element Binding Transcription Factor 2 (*SREBF2*) *A* allele in patients [159]. Of note, there were genes linked to the response to metformin, which had an effect on symptoms associated with metabolic syndrome. Transmembrane protein 18 (*TMEM18*) and Glucosamine-6-Phosphate Deaminase 2 (*GNPDA2*) minor allele carriers on metformin had a greater reduction in insulin levels and were more likely to lose more than 7% of their body weight after metformin treatment [158].

Blood disorders mainly presented in the form of agranulocytosis or granulocytopenia, which is a known possible severe side effect of clozapine. Elevated expression levels of the proapoptotic genes *TP53*, *Bax α* and *Bik* were found in patients experiencing agranulocytosis [152]. The Human Leukocyte Antigen (*HLA*) system encodes molecules necessary for pro-inflammatory responses, and several *HLA* polymorphisms were also linked to an increased risk of granulocytopenia/agranulocytosis in four separate studies [153,154,155,156]. Two other side effects were reported by the included studies, with Adrenoceptor α 2A (*ADRA2A*) *rs1800544 C/C* genotype being linked to sialorrhea [28]. *ADRA2A* receptors are hypothesized to play a role in the regulation of neurotransmitter release [160]. *GNB3 T825*, on the other hand, was associated with convulsive episodes [104].

## 4. Discussion

This review provided an updated summary of the evidence of specific genetic variants related to TRS, clozapine resistance and clozapine side effects. Extant evidence comprised of associations between TRS and clozapine resistance with genetic factors related to mainly dopaminergic and serotoninergic neurotransmitter systems. For example, TRS was associated with *rs4680* and *rs4818* within *COMT* and *rs1799978* within *DRD2,* and clozapine resistance was associated with *DRD3* polymorphisms and *CYP1A2* polymorphisms. In addition, clozapine-related weight gain was associated with *LEP*, *SNAP-25* genes, and agranulocytosis risk with *HLA*-related polymorphisms.

This study found that most genes related to TRS were mainly associated with dopaminergic systems and serotoninergic systems. This is understandable, as the proposed hypotheses for the neurobiological mechanisms underlying TRS include dopaminergic and serotoninergic dysregulation with inter-relationships with glutamatergic abnormality [8]. The dopamine supersensitivity hypothesis has been proposed as a potential etiology for TRS [161]. It is also acknowledged that not all patients with TRS display hyperdopaminergic activity, and some TRS cases can have normal dopamine regulation or hypodopaminergic activity [162]. Other neurotransmitters, such as glutamate and serotonin, also play a role in the etiology of TRS. For example, it has been reported that elevated glutamate levels may contribute to TRS [11]. There is also evidence that serotonin affects the clinical efficacy of antipsychotics [163]. The interactions between *SLC6A3* and the variable number tandem repeat polymorphism inside *SERT-in2* may also contribute to the development of TRS [31].

Genes related to other systems, such as the endocannabinoid, oxytocin and miRNA, were also found to be related to TRS in the current review, highlighting the complex genetic etiology of TRS. The endocannabinoid system has been reported to affect neurodevelopment [164], and changes in cannabinoid receptors in certain brain regions were found in patients with schizophrenia [165]. Oxytocin may be involved in negative symptoms of schizophrenia, such as social withdrawal and flattened affect, and certain genotypes related to oxytocin were associated with a response to antipsychotic drugs [32]. The expression changes of miRNA also played a significant role in the pathogenesis of schizophrenia [166]. Recently the expression levels of miRNA have been found to be different between TRS and non-TRS [48].

For clozapine resistance, *COMT* activity had been reported to be related to the clozapine-induced release of dopamine in the prefrontal area [167]. The gene–gene interaction between *DRD4* and *COMT* may also modulate the clinical response to clozapine in TRS [86]. Polymorphisms in serotonin receptors were also involved in clozapine response [168], and serotonin modulators may be used to augment the effects of antipsychotics in schizophrenia [169]. Of note, in a neuroimaging study, glutamate and glutamine levels were lower in clozapine-resistant TRS compared to clozapine-responsive TRS [12], suggesting a possible role of glutamate in clozapine treatment response.

Notably, the genetic variants related to *CYP* enzymes were associated with clozapine resistance. Clozapine is primarily metabolized in the liver by the *CYP450* enzymes. The main *CYP* enzyme involved in clozapine metabolism is *CYP1A2,* which is a potential determinant of clozapine dose requirement [170]. There are other *CYP* enzymes involved in clozapine metabolism, including *CYP2C19*, *CYP2D6* and *CYP3A4* [171]. *CYP1A2* and *CYP3A4* are mainly responsible for the demethylation of clozapine, while *CYP3A4* is involved in the N-oxidation of clozapine [172]. A few studies have shed light on the association between *CYP*-genetic polymorphisms and clozapine response. For example, a recent study has shown that *CYP2C19* polymorphisms influenced clozapine responses during the treatment of schizophrenia [27].

Several genetic variants, such as *LEP* and *HLA* polymorphisms, were reported to be associated with clozapine side effects, such as weight gain and agranulocytosis in the current review. Leptin, a hormone-regulating adipose tissue, is increased in the circulatory levels by clozapine, and this could lead to weight gain during clozapine treatment [173]. The *LEP AA* genotype has been associated with higher weight gain, and the ***A*** allele group may be related to a slow adaption response to environmental factors that are predisposed to weight gain [157]. Other than weight gain, neutropenia or agranulocytosis is another significant side effect of clozapine. One of the genetic risk factors for agranulocytosis are the *HLA* polymorphisms, which were associated with a range of immunogenetic phenomena, and specific *HLA* alleles may contribute to other adverse drug reactions, such as Stevens-Johnson Syndrome [174].

The emergence of genetic findings in major psychiatric disorders has inspired the development of Precision Psychiatry, which aims to provide personalized treatment approaches and acknowledges that a singular medication, dose, and treatment plan may not be effective for all patients with similar diagnoses [175]. The current diagnostic process for TRS requires the patient to try at least two different antipsychotics before the confirmation of TRS [17]. With the development of Precision Psychiatry, it is possible that TRS, clozapine resistance or potentially serious adverse effects for clozapine can be identified earlier, with suitable biological markers and managed accordingly. For example, to prevent life-threatening Stevens-Johnson syndrome among some Asians, HLA-B*1502 allele genotyping prior to the initiation of carbamazepine therapy in new patients of Asian ancestry is now considered the standard of care [176]. In addition, traditionally, the process of selecting an appropriate antipsychotic often involves a “trial and error” approach, where clinicians prescribe different antipsychotic at different dosages until an acceptable efficacy and tolerable side effects are achieved clinically. With the help of genetic technology, Precision Psychiatry may potentially guide more accurate and effective treatment. For example, a patient with risk genes suggesting clozapine resistance may be offered an alternative treatment, such as other pharmacotherapies or electroconvulsive therapy, to control the persistent psychotic symptoms. Though they are still developing, genetic markers in psychiatry offer a promising opportunity to complement other germane biological markers to improve precision in the clinical management of patients with schizophrenia.

There are several limitations in this review. First, the sample size varies widely across the studies, and the smaller sample sizes may limit the power to detect differences. Second, fewer studies compared the differences between TRS and clozapine resistance. For this review, a total of forty-two studies looked at TRS only (40.4%), fifty-three studies looked at resistance to or side effects from clozapine (51.0%), and only nine studies looked at both TRS and clozapine resistance (8.6%). Third, there is less correlation with clinical factors such as symptomatology, psychotropic drug treatment, and cognitive functioning. Fourth, there is a dearth of longitudinal studies and inter-relationships with clinical course. Fifth, despite the fact that genetic factors account for some variability in TRS, clozapine resistance and tolerability, other biological factors such as inflammation, oxidative stress, and neuronal and synaptic functioning may be contributory, as suggested by the genetic signals, thus making up the larger biosignature overall.

What are the implications for the future research on TRS? The historical candidate association studies have several limitations, including inadequate statistical power, inconsistent results, and false associations [177,178]. With the advancements of gene detection technology, there is promise of better elucidation of the underlying genetic architecture of TRS and clozapine resistance. Following the cost reduction in new genotyping technology, it has been pointed out that psychiatric genetics is shifting away from candidate association studies based on historical choices [179]. Genome-wide association studies (GWAS) adopt an atheoretical approach, have improved our understanding of the genetic basis of schizophrenia and other psychiatric disorders [57,180], and have the potential to examine common genetic variants related to TRS [181]. A combination of GWAS with fine mapping and functional genomic data has been conducted on schizophrenia [182] and can potentially unravel insights into the neurobiology of TRS and prioritize the genetic factors for further evaluation. More recent investigations have also applied novel techniques, such as whole exome sequencing (WES) and polygenic risk score (PRS) analysis, to further explore the multiple genetic variants contributing to disease risk. WES can capture rare types of genetic variants, but there is only a small number of such studies on schizophrenia [183]. PRS provides a weighted sum of the number of risk alleles in an individual, with higher scores indicating a higher genetic risk burden [184], and there is some evidence that a higher genetic risk burden indicates a higher likelihood of developing TRS [185]. The development of a PRS for TRS may eventually lead to a high predictive accuracy of TRS [186]. Future work may also want to focus on greater correlation between the genetic factors and clinical phenotype as well as studying the inter-relationships with other measures such as neuroimaging and cognitive and longitudinal outcome variables.

## 5. Conclusions

In conclusion, in view of the huge clinical and socio-economic burden associated with TRS, there is compelling reason to better understand the biological basis of TRS with the potential for identifying novel treatment targets to optimize clinical treatment. This review of the extant genetic studies in TRS provided insights into the complex genetic heterogeneity related to different neurotransmitter systems, neuronal development and cellular functioning. Conducting genome-wide investigations on substantial cohorts and performing replication studies with a well-selected panel of causal single nucleotide variants will advance our knowledge of TRS and clozapine pharmacogenomics. Further work, including replication in larger multi-site genetic studies, is needed to elucidate putative risk genes and the interactions between different genes and their correlations with relevant clinical factors such as psychopathology, psychosocial functioning, cognition and progressive changes with treatment over time in TRS and clozapine resistance.

## Figures and Tables

**Table 1:** Risk genes/polymorphisms for treatment-resistant schizophrenia.

System	Gene Biomarkers of TRS	Polymorphisms for TRS	Papers
Dopamine	*COMT*	*rs4680* (*Met* allele carriers-males only)	[29]
		*rs4680 A/A* genotype (females only)	[30]
		*rs4818 C/C* genotype (females only)	[30]
		*rs4818-rs4680* non *G-G/G-G* haplotypes (females only)	[30]
	*COMT + GAD1*	*rs4680/rs3749034 Met(+)/T(𢄡)*	[25]
	*SLC6A3 + SERT-in2*	*SLC6A3 10/10* or *10/12* genotype + *SERT-in2 ls* or *ss* genotype	[31]
	*DRD2*	*rs1799978*	[32]
		*rs1799978* (*G* allele)	[33]
	*DDC*	*rs10499696* (*A/A* genotype)	[32]
	*COMT + DRD3*	*COMT rs4680 Met/Met* + *DRD3 rs6280* (*Ser/Gly*)	[33]
Serotonin	*5HT2A*	*2/2* genotype-males only	[34]
	*HTR2C*	*rs6318 Ser* non-carriers (males only)	[35]
		*rs3813929-rs6318* non *C-Ser* haplotypes (males only)	[35]
	*SERT-in2*	*SERT-in2 ll* genotype + *SLC6A3 9/10*, *9/11*, *9/9* and *6/6* genotype carriers	[31]
	*SERT-PR*	*SS* genotype	[31]
GABA/Glutamatergic	*GAD1*	*rs3749034*	[26]
	*GABBR2*	*rs10985765*	[26]
	*GRM3*	*rs1989796* (*TT* genotype)	[36]
		*rs1476455* (*CC* genotype)	[36]
Endocannabinoid	*CNR1*	*rs806368* (*T/C* and *T/T* genotypes)	[32]
		Increased expression	[37]
		*rs1049353 G/G* genotype	[32]
Oxytocin	*OXT*	*rs2740210* (*C* allele)	[32]
	*OXTR*	*rs2228485* (*A* allele, *A/A* genotype)	[32]
Others	*AKT1*	Upregulated	[37]
	*BDNF*	Higher expression	[38]
		*rs10767665* (minor allele)	[39]
		*rs11030104* (minor allele)	[39]
		*rs6265* (minor allele)	[39]
		*rs10501087* (minor allele)	[39]
		*rs11030096* (minor allele)	[39]
		*rs4923460* (minor allele)	[39]
		*rs6416056* (minor allele)	[39]
		*rs10742178* (minor allele)	[39]
		*rs4922788* (minor allele)	[39]
		*rs1114029* (minor allele)	[39]
		Higher expression of *BDNF*-associated lncRNA *PKNY*	[38]
	*CYP2A6*	Higher mRNA transcript levels	[40]
	*CYP2D6*	Reduced mRNA expression	[40]
	*CYP3A4*	Reduced mRNA expression	[40]
		LoF variant carrier	[41]
	*DICER1*	Upregulated	[37]
	*GRB10*	*rs2237457 T/T* genotype	[42]
	*GSTT1 + GSTM1*	Double-null (𢄡/𢄡) genotype	[43]
	*MAPT*	Lower methylation rates	[44]
	*MBL2*	*AA* and *BB* genotypes	[45]
	miRNA	Increased expression of *miR-181b-5p*, *miR-195-5p* and *miR-301a-3p*	[46]
		Homo sapiens (*hsa*)-*miR-218-5p* and *hsa-miR-1262* upregulated	[47]
		*hsa-miR-675-3p* expression upregulated	[48]
	*NRG1*	*rs7834206*	[49]
	*NRXN1*	Lower methylation rates	[44]
	*P70S6K*	Elevated	[50]
	Reelin	*(CGG)_10_* allele and genotypes	[51]
	*SNAP-25*	*MnlI* polymorphism, *T/G* or *G/G* genotype	[52]
		*TaiI* polymorphism, *T/T* or *T/C* genotype	[52]
	*TNF-α − 238*	*GG*	[53]
	*UBE2K*	Higher mRNA levels	[54]
	*UFD1*	Increased expression	[37]

**Table 2:** Risk genes/polymorphisms for Clozapine resistance.

System	Gene Biomarkers of CLZ Resistance	Polymorphisms for CLZ Resistance	**Papers**
Dopamine	*DRD2*	*rs1799978* (*T* allele)	[32]
		*rs2514218* (*G/G*)	[81]
		*TaqIB B1 C* allele in African-Americans only	[82]
	*DRD3*	*Ser9* allele	[83]
		*Ser9/Ser9* genotype	[83]
		*1-1* genotype	[84]
	*DRD4*	*120-bp* tandem repeat polymorphism 1-copy allele—in African Americans only	[85]
		*(G)n 142-bp/140-bp* genotype	[85]
		*48-bp* repeat polymorphism non-*4R* alleles (homozygous carriers)—in Caucasians only	[85]
		*(G)n 142-bp* allele-*rs11246226 A* allele haplotype—in Caucasians only	[85]
		*(G)n 142-bp* allele-*rs936465 C* allele haplotype—in Caucasians only	[85]
		*(G)n rs11246226 A* allele-*rs936465 C* allele haplotypes—in African Americans only	[85]
	*COMT*	*rs4680* (*Met* allele carrier)	[33]
	*COMT + DRD4*	*COMT val/val* + *DRD4 120/120* or *120/240*	[86]
	*SLC6A3*	*rs2975226-71A* allele	[87]
		*rs2652511 (T-844C)* x *rs2975226 (T-71A)* x *rs2963238 (Intron 1: A1491C)* Haplotype *C-A-C*	[87]
Serotonin	*5HT2A*	*tyr452* allele	[88]
	*HTR2C*	*rs6318 Ser* non-carrier (males only)	[35]
	*HTR3A*	*rs1062613* (*C* allele; *T/T* genotype)	[89,90]
		*rs2276302* (*A* allele)	[89]
	*HTR3B*	*–100_–102delAAG* polymorphism and *–100_–102delAAG(del)* minor allele	[91]
	*SLC6A4/HTTLPR*	*rs25531* (*S*′-allele)	[92]
		*rs25531 S*′/*S*′ homozygous or *S*′/*L*′ heterozygous	[92]
GABA/Glutamatergic	*GAD1*	Higher mRNA levels	[93]
	*GAD25*	Higher mRNA levels	[93]
	*GAD67*	Higher mRNA levels	[93]
Endocannabinoid	*CNR1*	*rs806379* (*C* allele)	[32]
		*rs1043953* (*A* allele)	[32]
Others	*ABCG2/BCRP*	*421 C/C* genotype	[94]
	*BDNF*	Higher expression	[95]
		*Val/Val* genotype	[96]
	*CREBBP*	CpG site *cg05151055* (decreased methylation)	[97]
	*CYP1A2*	*1F (*AA* and *AC* genotypes)	[98]
		*-163A* allele carriers (smokers only)	[99]
		Higher activity score	[100]
	*CYP2C19*	Lower activity score	[100]
		*1/*17 genotype	[27]
		*2 allele	[27]
	*CNTNAP2*	Higher mRNA levels	[93]
	*DISC1*	*rs3738401* (minor allele *A*)	[101]
		*rs3738401* (*A/A* or *A/G* genotypes)	[101]
		*rs6675281* (*T* allele non-carriers)	[101]
	*DTNBP1*	*rs742105* (allele *C*)—in African-Americans only	[102]
		*rs742105* (genotype *C/C*)—in African-Americans only	[102]
	*GFRA2*	*1-1-2 SNP27-SNP34-SNP37* haplotype non-carriers	[103]
	*GNB3*	*825* (*T/T* genotype)	[104]
	*IMPA2*	Higher mRNA levels	[93]
	*KCNJ10*	Lower expression	[95]
	*LNX1*	Higher expression	[95]
	*NFIB*	*rs28379954* (*C* allele)	[105]
	*NRXN1*	*rs1045881* (*T* allele)	[106]
	*SERPINA5*	Lower expression	[95]
	*TET1*	Lower mRNA levels	[93]
	*TMCC3*	Higher expression	[95]
	*SLC25A27/UCP4*	Non-carriers of *CCAC* haplotype of *rs3757241*, *rs10807344*, *rs9395206*, and *rs2270450*	[107]

**Table 3:** Genes/polymorphisms associated with Clozapine side effects.

Type of Side Effect	Gene Biomarkers Associated with Side Effects	Polymorphisms for Side Effects	Papers
Low High-density lipoprotein	*DRD2*	*141 Ins C* allele homozygous genotype	[151]
Sialorrhea	*ADRA2A*	*rs1800544* (*C/C* genotype)	[28]
Agranulocytosis	*Bax α*	Elevated expression levels	[152]
Agranulocytosis	*Bik*	Elevated expression levels	[152]
Agranulocytosis	*TP53*	Elevated expression levels	[152]
Convulsive episode	*GNB3*	*T825* carriers	[104]
Agranulocytosis	*HLA*	*Cw7-B18* and *Cw7-B39*	[153]
Agranulocytosis	*HLA*	DRB5*0201-DRB4*000	[153]
Agranulocytosis	*HLA*	Cw7-B18-DRB5*000, Cw7-B39-DRB5*000, and Cw7-B44-DRB5*000	[153]
Granulocytopenia/agranulocytosis	*HLA*	*DQw2*	[154]
Granulocytopenia/agranulocytosis	*HLA*	DQB1*0201	[154]
Agranulocytosis	*HLA*	*B38*—in Ashkenazi only	[155]
Agranulocytosis	*HLA*	*B38*, *DR4*, *DQw3* haplotype	[156]
Weight gain	*LEP-2548A/G*	*A/A* genotype	[157]
Weight gain	*LEP-2548A/G*	*G/A* or *G/G* genotypes	[122]
Weight gain	*SNAP-25*	*MnlI* polymorphism, *T/T* genotype	[52]
Weight gain	*SNAP-25*	*TaiI* polymorphism, *C/C* genotype	[52]
Higher blood pressure	*SH2B1*	Minor allele carriers	[158]
Metabolic syndrome	*SREBF2*	*A* allele	[159]
Greater reduction in insulin levels	*TMEM18*	Minor allele carriers (*TT* + *CT* genotypes) + on metformin	[158]
More likely to lose more than 7% of body weight after metformin treatment	*TMEM18*	Minor allele carriers (*TT* + *CT* genotypes) + on metformin	[158]
More likely to lose more than 7% of body weight after metformin treatment	*GNPDA2*	Minor allele carriers (*AG* + *GG* genotypes) + on metformin	[158]

## Data Availability

Details of included studies are found in Supplementary Table S1.

## Supplementary Materials

The following supporting information can be downloaded at: https://www.mdpi.com/article/10.3390/genes14030689/s1, Table S1: Summary of study and population characteristics, definition of treatment resistance, and correlates.
