# Supplementary material for: Treatment-Resistant Schizophrenia, Clozapine Resistance, Genetic Associations, and Implications for Precision Psychiatry: A Scoping Review
Source: Genes (Basel). 2023 Mar 10;14(3):689. doi: 10.3390/genes14030689 (PMC10048540; doi:10.3390/genes14030689)
Supplement: Supplementary file 1 [file genes-14-00689-s001.zip › genes-2240259-supplementary.pdf]

Supplementary Table S1. Summary of study and population characteristics, definition of treatment resistance, and correlates

Author (Year), Country, Journal	Population characteristics (sample size, age, gender, DOI)	Definition of TRap	Definition of response to CLZ	Genes of interest
Aytac et al. (2022)<br><br>Turkey<br><br>Immunol Invest	n = 113 (SCZ, n = 25 TRap, n = 88 treatment responsive)<br>n = 107 (HC)<br><br>40.5 ± 10.4 years (SCZ)<br>Age not provided for HC<br><br>74.3% male (SCZ)<br>Gender not provided for HC<br><br>DOI not provided	A lack of response to treatment and the persistence of moderate-to-severe symptoms despite the usage of two different APs in the appropriate dose (400–600 mg/day chlorpromazine) and duration (4–6 weeks)	---	TNF-α − 238 GG genotype distribution (higher in TRap vs. treatment responsive SCZ)

Funahashi et al. (2022)<br><br>Japan<br><br>World J Biol Psychiatry	n = 9 (TRap on CLZ)<br>n = 9 (HC)<br><br>41.9 ± 9.4 years (TRap on CLZ)<br>41.9 ± 9.4 years (HC)<br><br>44.4% male (TRap on CLZ)<br>44.4% male (HC)<br><br>22.4 ± 8.5 years (DOI for TRap on CLZ)<br><br>Replication set<br><br>n = 50 (HC)<br>n = 50 (non-TRap)<br><br>55.1 ± 16.4 years (HC)<br>55.1 ± 15.7 years (non-TRap)<br><br>48.0% male (HC)<br>48.0% male (non-TRap)<br><br>30.3 ± 14.9 years (DOI for non-TRap)	Those who did not respond to more than two sufficient doses of APs (more than 600 mg/day of chlorpromazine equivalent), including at least one atypical AP for more than 4 weeks	---	hsa-miR-675-3p expression (higher in TRap vs. HC)
Hribkova et al. (2022)<br><br>Czechia, Europe<br><br>Front Cell Neurosci	n = 6 (SCZ, n = 3 CLZ-responsive, n = 3 CLZ-resistant)<br>n = 2 (HC)<br><br>39.3 years (SCZ)<br><br>100.0% male (both SCZ and HC)<br><br>13.5 years (DOI for SCZ)	---	CLZ resistance: (1) the patient had to be treated either with a combination of CLZ and another AP, or with another AP medication alone; (2) during the course of the disorder, the patient had to show either no effect of CLZ or its combination with another medication	Genes associated with retrograde endocannabinoid signaling, morphine addiction, and GABAergic and cholinergic synapses (downregulated in CLZ responsive vs. HC)<br><br>Na+ channel genes (SLC4A4, SCL32A1, SLC13A4, SLC1A4, SLC17A8, SCN2A, ATP1B1, SCN3A, ATP1A2, ATP1A3, SLC6A1, HCN4, SLC17A6) (downregulated in CLZ responsive vs. CLZ non-responsive)<br><br>K+ channel genes (KCNK10, KCNB1, KCNH8, KCTD2, ATP1B1, KCNQ2, ATP1A2, ATP1A3, TMEM38A, KCNG1,

			on major SCZ symptoms, or a relapse of symptoms during the CLZ treatment that necessitated acute treatment	KCNF1, KCNJ4, HCN4) (downregulated in CLZ responsive vs. CLZ non-responsive)
Miyazawa et al. (2022)<br><br>Japan<br><br>Mol Biol Rep	n = 226 (TRap)<br>n = 681 (non-TRap)<br>n = 508 (HC)<br><br>48.4 ± 13.0 years (TRap)<br>44.4 ± 15.0 years (non-TRap)<br>36.8 ± 14.7 years (HC)<br><br>47.3% male (TRap)<br>50.4% male (non-TRap)<br>57.1% male (HC)<br><br>DOI not provided	No sufficient improvement in positive symptoms with sufficient doses of two or more trials of APs (chlorpromazine-equivalent dose of 600 mg or higher) for a sufficient duration (4 weeks or longer) in each trial	---	---
Okhuijsen-Pfeifer et al. (2022)<br><br>Netherlands, Germany, Austria, Finland, Australia, Turkey<br><br>Transl Psychiatry	Recruited from different places (All SCZ on CLZ)<br><br>CLOZIN consortium<br>n = 407<br>43.2 ± 11.9 years<br>70.5% male<br><br>GROUP consortium<br>n = 152<br>27.6 ± 5.6 years<br>83.4% male<br><br>CRC cohort<br>n = 67<br>41.6 ± 9.8 years<br>71.6% male<br><br>Hacettepe University cohort	Had a primary diagnosis of schizophrenia spectrum disorder, and were using CLZ	---	Higher CYP2C19 activity score (greater probability of low symptom severity/symptom improvement and lower symptom severity scores)<br><br>Higher CYP1A2 activity score (lower dose-adjusted CLZ levels)

	n = 34<br>44.1% male<br>42.5 ± 7.7 years<br><br><br>MHS Rivierduinen cohort<br>n = 24,<br>40.9 ± 12.6 years<br>91.7% male<br><br>DOI not provided			
Sun et al. (2022)<br><br>China<br><br>Biol Psychiatry	RNA-seq cohort:<br><br>n = 7 (n = 3, TRap OLA responders, n = 4 OLA non-responders)<br><br>28.8 ± 7.4 years (male)<br>19.7 ± 5.5 years (female)<br><br>57.1% male<br><br><br>Genotyping cohort:<br><br>n = 356 (n = 202 OLA responders, n = 154 OLA non-responders)<br><br>39.8 ± 14.4 years (male)<br>40.4 ± 14.1 years (female)<br><br>50.6% male<br><br>DOI not provided	Inpatients with TRap who were undergoing OLA monotherapy were recruited in this study<br><br>TRap inpatients whose PANSS scores reduced by more than 25% at 8-week trials of OLA monotherapy were considered as OLA responder patients, whereas TRap inpatients whose PANSS scores decreased <25% were considered as OLA non-responder patients	---	The NRG1 rs7834206 genotype (AA vs. CC: odds ratio = 0.5813, 95% CI = 0.3768–0.8969, p = .01346) was found to be significantly associated with OLA treatment response
Talarico et al. (2022)<br><br>Brazil<br><br>Mol Neurobiol	n = 63 (TRap, n = 51 on CLZ)<br>n = 111 (non-TRap)<br><br>34.0 ± 9.3 years (TRap)<br>37.7 ± 10.5 years (non-TRap)<br><br>66.7% male (TRap)<br>71.2% male (non-TRap)	Unresponsive to at least two APs used in monotherapy for a period of 4 to 6 weeks with appropriate doses of the drug and to present moderate to severe psychopathology and persistence of positive symptoms	---	PTPRD, KALRN, FHIT, DCC, FSTL5, TEK, DNM3, PITPNA, ERBB4 (related to TRap)<br><br>SVIL, NRG1, and ESR1 (upregulated by CLZ)

	DOI not provided			
Zazueta et al. (2022)<br><br>Chile<br><br>Int J Neuropsychopharmacol	n = 135 (TRap on CLZ, n = 37 CLZ responsive, n = 22 CLZ refractory)<br>n = 61 (second generation AP)<br>n = 80 (HC)<br><br>41.4 years (TRap)<br>27.7 years (second generation AP)<br>26.2 years (HC)<br><br>68.8% male (TRap)<br>72.1% male (second generation AP)<br>33.7% male (HC)<br><br>DOI not provided	Having failed at least 2 adequate trials of AP including at least 1 atypical AP drug, and currently receiving at least 300 mg of CLZ for at least 6 months	Remitted (i.e., responsive to CLZ): Mild or lower score on BPRS items 4, 7, 8, 11, 12, 15, and 16<br><br>Refractory (i.e.,CLZ-resistant): Global score ≥45 on the 18-item BPRS or a score ≥4 on 2 or more psychotic symptoms	OXTR rs2228485 G/G genotype, CNR1 rs806368 C/C genotype, CNR1 rs1049353 A/A genotype, and DDC rs10499696 G/G genotype (lower risk of TRap)<br>OXTR rs2228485 A/G genotype, CNR1 rs1049353 G/A genotype, and DDC rs10499696 A/G genotype (lower risk of TRap)<br>OXT rs2740210 C-allele and OXTR rs2228485 A-allele (TRap)<br>Differences in allele frequencies for OXT rs877172, OXTR rs22228485, CNR1 rs806368, CNR1 rs1049353, CNR1 rs806379, CNR1 rs806380, DDC rs11283133, DDC rs10499696, DRD2 rs1799978 (TRap vs. non-TRap)<br><br>Frequency of CNR1 rs806379 C-allele and rs1043953 A-allele (higher in CLZ refractory vs. CLZ responsive)<br>Frequency of DRD2 rs1799978 G-allele (lower in CLZ refractory vs. CLZ responsive)
Akkouh et al. (2021)<br><br>Czechia, Europe<br><br>Schizophr Bull	n = 6 (SCZ on CLZ, n = 3 CLZ responders, n = 3 CLZ non-responders)<br><br>39.3 years (All SCZ)<br><br>100.0% male<br><br>13.5 years (DOI for all SCZ on CLZ)	Not provided, but all patients were on CLZ	(1) they were treated with any other AP alone or in combination with CLZ at the initial assessment, and (2) showed either no effect of CLZ or its combination with other medications, or experienced relapse of the symptoms during the CLZ treatment necessitating	Expression levels of SYT1 and BDNF (lower in CLZ responders vs. CLZ non-responders)<br>Expression level of KCNJ10 (higher in CLZ responders vs. CLZ non-responders)<br>Expression level of SERPINA5 (higher in CLZ responders vs. CLZ non-responders in global Differential Expression analysis)<br>Expression level of LNX1, TMCC3, and ADAMTS9 (lower in CLZ responders vs. CLZ non-responders in global Differential Expression analysis)

			acute treatment with other drugs	
Ammar et al. (2021)<br><br>Tunisia<br><br>Pharmacogenomics J	n = 51 (SCZ with TRap on CLZ)<br><br>34.8 ± 8.7 years<br><br>78.4% male<br><br>DOI not provided	Not provided, but all patients were on CLZ	---	CYP1A2*1 F polymorphism - AA and AC genotypes (lower C0 and C0/D vs. CC genotype)
Aytac et al. (2021)<br><br>Turkey<br><br>Neurosciences Riyadh	n = 118 (SCZ, n = 26 TRap, n = 92 non-TRap)<br><br>41.0 years<br><br>73.7% male<br><br>15.5 years (DOI for all SCZ)	A lack of response to treatment and the permanence of moderate to severe symptoms despite 2 separate APs in the proper dose (400-600 mg/day chlorpromazine) and duration (4-6 weeks)	---	Frequency of MBL2 AB genotype (higher in non-TRap vs. TRap)
Badrlou et al. (2021)<br><br>Iran<br><br>J Mol Neurosci	n = 50 (SCZ on CLZ)<br>n = 50 (HC)<br><br>51.3 ± 10.4 years (male, SCZ on CLZ)<br>46.6 ± 7.4 years (female, SCZ on CLZ)<br>50.0 ± 12.8 years (male, HC)<br>50.0 ± 12.8 years (female, HC)<br><br>66.0% male (SCZ on CLZ)<br>66.0% male (HC)<br><br>16.7 ± 9.6 years (DOI for male, SCZ on CLZ)<br>11.5 ± 6.1 years (DOI for female, SCZ on CLZ)	Not provided, but all patients were on CLZ	---	Relative expression of PNKY (higher in SCZ on CLZ vs. HC)<br>BDNF expression (higher in SCZ on CLZ vs. HC)

Jahn et al. (2021)<br><br>France<br><br>Neuropsychobiology	n = 28 (TR SCZ not consuming THC)<br>n = 22 (TR SCZ consuming THC)<br>n = 46 (HC)<br><br>35.6 ± 10.6 years (TR SCZ not consuming THC)<br>28.2 ± 7.1 years (TR SCZ consuming THC)<br>32.9 ± 10.4 years (HC)<br><br>73.8% male (TR SCZ not consuming THC)<br>85.2% male (TR SCZ consuming THC)<br>84.6% male (HC)<br><br>DOI not provided	Nonresponse to at least two different dopaminergic AP drugs, administered in a high enough concentration and for adequate duration		Methylation rate of NRXN1 (Lower in TR SCZ not consuming THC vs. HC)<br>Methylation of NRXN1 promoter (Two times higher in TR SCZ consuming THC vs. TR SCZ non-consumers)<br><br>Methylation rate of MAPT (Lower in TR SCZ not consuming THC vs. HC)
Kogure et al. (2021)<br><br>Japan<br><br>J Mol Neurosci	n = 171 (TRap)<br>n = 592 (non-TRap)<br>n = 447 (HC)<br><br>49.7 ± 13.0 years (TRap)<br>45.0 ± 15.4 years (non-TRap)<br>36.4 ± 15.1 years (HC)<br><br>50.3% male (TRap)<br>47.4% male (non-TRap)<br>57.2% male (HC)<br><br>DOI not provided	Never having shown a sufficient response (i.e., <40 points on GAF over the past 12 months) to at least two types of APs with each chlorpromazine-equivalent dose of >600 mg for >4 weeks	---	Percentage of subjects with COMT rs4680/GAD1 rs3749034 Met(+)/T(-) (higher in the TRap vs. non-TRap and HC)
Krzystanek et al. (2021)<br><br>Poland<br><br>Pharmacol Rep	n = 45 (CLZ-resistant SCZ)<br>n = 30 (HC)<br><br>49.1 ± 9.9 years (CLZ-resistant SCZ)<br>57.0 ± 7.3 years (HC)<br><br>55.6% male (CLZ-resistant SCZ)<br>53.3% male (HC)		Treated with CLZ at a therapeutic dose for at least 3 months without improvement	---

	23.1 ± 11.5 years (DOI for CLZ-resistant SCZ)			
Nakata et al. (2021)<br><br>Japan<br><br>J Psychiatr Res	n = 28 (SCZ in remission)<br>n = 30 (TRap)<br><br>39.5 ± 8.5 years (SCZ in remission)<br>43.8 ± 10.4 years (TRap)<br><br>53.6% male (SCZ in remission)<br>53.3% male (TRap)<br><br>15.0 ± 7.7 years (DOI for SCZ in remission)<br>22.7 ± 10.0 years (DOI for TRap)	Two different chemical classes of AP unable to sufficiently relief positive symptoms with sufficient dosage (chlorpromazine equivalent dose of >600 mg/day) for >4 weeks, and the patient did not exceed the mean of 41 points of GAF within 1 year	---	---
Regen et al. (2021)<br><br>Germany<br><br>Mol Psychiatry	n = 10 (SCZ on CLZ)<br>n = 10 (SCZ on other medications)<br>n = 10 (HC)<br><br>42.2 ± 11.1 years (SCZ on CLZ)<br>42.5 ± 13.9 years (SCZ on other medications)<br>42.2 ± 11.3 years (HC)<br><br>50.0% male (SCZ on CLZ)<br>50.0% male (SCZ on other medications)<br>50.0% male (HC)<br><br>DOI not provided	A clinical DSM-5 diagnosis of SCZ more than five years prior to inclusion were treated in our clinic as in- or outpatients and were on a stable medication with clozapine (N = 10) or other APs (N = 10) (did not mention TRS)	---	Whole blood mRNA expression of at-retinoic acid (RA)-catabolizing and at-RA-inducible CYP26A (reduced in patients without CLZ vs. SCZ on CLZ)<br><br>mRNA levels of RA-inducible gene STRA6 (reduced in SCZ without CLZ vs. HC)<br><br>mRNA expression of CYP2D6 and 3A4 (reduced in SCZ on CLZ vs. HC)
Sershen et al. (2021)<br><br>USA<br><br>Biomark Neuropsychiatry	n = 61 (SCZ, n = 28 on CLZ)<br>n = 49 (HC)<br><br>44.7 ± 9.7 years (SCZ)<br>35.6 ± 10.8 years (HC)	Not all patients are TRap. Some were on CLZ	---	GAD1, GAD67, GAD25, CNTNAP2, and IMPA2 mRNAs (higher in SCZ on CLZ vs. SCZ not on CLZ)<br>TET1 mRNAs (lower in SCZ on CLZ vs. SCZ not on CLZ)

	85.2% male (SCZ)<br>67.3% male (HC)<br><br>DOI not provided			
Rodrigues-Silva et al. (2020)<br><br>Brazil<br><br>Neuropsychiatr Dis Treat	n = 63 (TRap)<br>n = 45 (CLZ non-responders)<br><br>40.0 ± 11.0 years (TRap)<br>39.0 ± 9.0 years (CLZ non-responders)<br><br>68.3% male (TRap)<br>55.6% male (CLZ non-responders)<br><br>DOI not provided	1) at least three periods of treatment in the preceding five years with neuroleptic agents (from at least two different chemical classes) at dosages equivalent to or greater than 1000 mg/d of chlorpromazine for a period of six weeks, each without significant symptomatic relief, and (2) no period of good functioning within the preceding five years	Patients were classified as TRS (n = 63) or SRS (super refractory schizophrenia) (n = 45) following criteria described by Kane et al (1988) and de Brito et al (2015)	CYP2C19*1/*17 (greater frequency in CLZ non-responders vs. TRap and HC)<br>CYP2C19*17 (lower BPRS scores/better clinical response in TRap)<br>CYP2C19*2 (lower CLZ dosages in TRap)<br>CYP2C19*2 (lower BPRS scores/better clinical response in CLZ non-responders)
Smith et al. (2020)<br><br>Norway<br><br>Transl Psychiatry	n = 484 (TRap on CLZ)<br><br>37.0 years<br><br>61.6% male<br><br>DOI not provided	Determined by therapeutic drug monitoring (TDM) of CLZ, with the applied therapeutic reference range being 300–2500 nmol/L	---	NFIB rs28379954 C allele carriers (higher likelihood of having CLZ serum concentrations below therapeutic range vs. TT allele carriers regardless of smoking status)<br><br>NFIB rs28379954 C allele carriers (lower CLZ dose-adjusted serum concentration vs. TT genotype carriers regardless of smoking status)<br><br>NFIB rs28379954 C allele carriers (lower CLZ dose-adjusted serum concentration of N-desmethylclozapine vs. TT genotype carriers regardless of smoking status)<br><br>NFIB rs28379954 TT allele carriers who are smokers (higher CLZ-to-N-desmethylclozapine ratio vs. C allele carriers who are smokers)
You et al. (2020)<br><br>China<br><br>Mol Med Rep	n = 34 (TRap)<br>n = 31 (HC)<br><br>41.8 ± 11.4 years (TRap)	Patients who only have a minor or no response to at least two non-CLZ drugs at adequate doses and treatment cycles are diagnosed with TRap according to	---	Expression of Homo sapiens (hsa)-miR- 218-5p and hsa-miR- 1262 (upregulated in TRap vs. HC)

	37.1 ± 11.4 years (HC)<br><br>50.0% male (TRap)<br>51.6% male (HC)<br><br>DOI not provided	the guidelines of the American Psychiatric Association		
Cavieres et al. (2019)<br><br>Chile<br><br>Schizophr Res Treatment	n = 100 (SCZ on CLZ; n = 41 CLZ-resistant, n = 24 CLZ responsive)<br>n = 48 (HC)<br><br>42.7 ± 11.5 years (SCZ on CLZ)<br>26.2 ± 15.3 years (HC)<br><br>65.0% male (SCZ on CLZ)<br>48.0% male (HC)<br><br>DOI not provided	1) Failed to improve after two courses of APs at adequate doses for 6 to 8 weeks, with at least one of them of second generation<br>2) Have been receiving CLZ at a minimum dose of 300 mg per day, for at least six months	Overall score ≥ 45 on the BPRS scale and/or ≥ 4 (moderate) on two or more items of psychotic symptoms, after at least six months of treatment, with doses ≥ 300 mg of CLZ	---
Escamilla et al. (2019)<br><br>Mexico<br><br>Neuropsychiatr Dis Treat	n = 88 (Treatment-responsive SCZ)<br>n = 49 (SCZ with TRap)<br>n = 33 (Ultra treatment-resistant SCZ)<br><br>38.3 ± 10.3 years (Treatment-responsive SCZ)<br>37.9 ± 9.8 years (SCZ with TRap)<br>43.6 ± 12.8 years (Ultra treatment-resistant SCZ)<br><br>68.0% male (Treatment-responsive SCZ)<br>65.0% male (SCZ with TRap)<br>58.0% male (Ultra treatment-resistant SCZ)<br><br>DOI not provided	Documented clinical history of treatment failure with at least 2 APs (minimum doses of 600 mg/d of chlorpromazine equivalents, for at least 6 weeks) but with a response for CLZ	Failed at least two trials of APs and a trial of CLZ (at least for 6 months, within a dose range between 250 and 600 mg/d)	Frequency of DRD2 rs1799978 G allele (higher in TRap vs. ultra treatment-resistant SCZ and treatment responsive SCZ)<br>COMT rs4680 Met/Met + DRD3 rs6280 Ser/Gly (predictive of resistant-to-treatment phenotype)<br>Frequency of COMT rs4680 met allele carriers (higher in ultra treatment-resistant SCZ vs. treatment responsive SCZ)
Hajj et al. (2019)<br><br>Lebanon	n = 100 (SCZ; 53 TRap, 47 treatment responsive)	PANSS score was ≥4 in at least two of categories: P2 (conceptual disorganization), P3 (hallucinatory behavior), P6		COMT rs4680 Met allele carriers (increased risk of TRap based on BPRS or PANSS vs. Val/Val for males only)

Int J Mol Sci	49.4 ± 12.6 years (All SCZ)<br><br>27.0% male (All SCZ)<br><br>DOI not provided	(suspiciousness/persecution), G9 (unusual thought content) (used as a secondary end-point)		
Li et al. (2019)<br><br>USA<br><br>Front Psychiatry	n = 171<br><br>78.0% on CLZ<br>7.0% on melperone<br>3.8% on risperidone<br>2.1% on olanzapine<br>9.0% on typical antipsychotics<br><br>Age not provided<br><br>67.3% male<br><br>DOI was stratifed by gender and genotype	Reduction of >20% in the BPRS total score or subcategories, BPRS Positive and Negative	Reduction of >20% in the BPRS total score or subcategories, BPRS Positive and Negative	HTR2C rs6318 Ser carrier (AP treatment positive and negative symptom improvement at 6 months in male SCZ only)<br>HTR2C rs6318 Ser carrier (CLZ treatment positive and negative symptom improvement at 6 months in male SCZ only)<br>HTR2C rs6318 Ser carrier (CLZ treatment negative symptom improvement at 6 weeks in female SCZ only)<br><br>HTR2C rs3813929-rs6318 C-Ser haplotype (AP treatment positive and negative symptom improvement at 6 months in male SCZ only)
Martínez-Magaña et al. (2019)<br><br>Mexico<br><br>Rev Invest Clin	n = 12 (SCZ)<br>n = 7 (non SCZ)<br><br>Age not provided<br><br>Gender not provided<br><br>DOI not provided	Nonresponse to at least two trials of AP medication of adequate dose and duration		Carrier of the loss-of-function variant in CYP3A4 (a patient with this variant had TRap)
Meiklejohn et al. (2019)<br><br>Australia<br><br>J Psychiatr Res	Clinical cohort<br><br>n = 71 (TRap)<br>n = 57 (HC)<br><br>40.2 ± 9.7 years (TRap)<br>9.5 ± 10.7 years (HC)<br><br>74.6% male (TRap)<br>61.4% male (HC)	Poor functioning and residual symptoms despite adequate trials of at least two APs before management with CLZ	---	Elevated levels of UBE2K mRNA (in whole blood of TRap SCZ vs. HC)

	17.1 ± 8.1 years (DOI for TRap)<br><br>Post mortem brain cohort<br><br>n = 37 (TRap)<br>n = 37 (HC)<br><br>51.2 ± 14.1 years (TRap)<br>51.1 ± 14.6 years (HC)<br><br>64.9% male (TRap)<br>81.1% male (HC)<br><br>27.6 ± 13.8 years (DOI for TRap)			
Chhabra et al. (2018)<br><br>India<br><br>Acta Neuropsychiatr	n = 32 (SCZ with TR auditory hallucinations)<br><br>35.8 ± 12.3 years<br><br>50.0% male<br><br>DOI not provided	Persistent auditory hallucinations (Psychotic Symptom Rating Scale) despite adequate AP treatment were administered with an add-on tDCS		COMT-GG and the co-occurrence of NRG1- AA genotype (better response to tDCS and improvement in auditory verbal hallucinations)
Chau et al. (2018)<br><br>Australia<br><br>Prog Neuropsychopharmacol Biol Psychiatry	n = 71 (TRap)<br>n = 30 (Recent-onset SCZ)<br>n = 57 (HC)<br><br>40.0 ± 10.0 years (TRap)<br>21.0 ± 2.0 years (Recent-onset SCZ)<br>40.0 ± 11.0 years (HC)<br><br>75.0% male (TRap)<br>77.0% male (Recent-onset SCZ)<br>61.0% male (HC)<br><br>17.0 ± 8.0 years (DOI for TRap)<br>1.1 ± 1.0 years (DOI for recent-onset SCZ)	Failed to response to two or more previous trials of APs with persistent symptoms and poor functioning	---	SELENBP1 mRNA levels (positive correlation with CLZ plasma levels and duration of illness in TRap only)
Moretti et al. (2018)	n = 78 (SCZ with TRap)<br>n = 84 (SCZ non-TRap)	No response to at least two APs used in monotherapy for a	---	CNR1 and UFD1L gene expression (TRap and non-TRap > HC)

Brazil<br><br>Mol Neurobiol	n = 94 (HC)<br><br>38.2 ± 10.5 years (TRap)<br>39.4 ± 11.2 years (non-TRap)<br>35.9 ± 11.4 years (HC)<br><br>64.1% male (TRap)<br>61.9% male (non-TRap)<br>56.4% male (HC)<br><br>16.3 ± 7.8 years (DOI for TRap)<br>14.3 ± 9.4 years (DOI for non-TRap)	minimum period of 4 to 6 weeks with appropriate doses of the drug and presence of moderate to severe psychopathology, especially positive symptoms		AKT1 and DICER1 gene expression (upregulated in TRap vs. HC)
Pinto et al. (2018)<br><br>Brazil<br><br>Rev Lat Am Enfermagem	n = 72 (SCZ with TRap on CLZ)<br><br>Approximately 43 years<br><br>50.0% male<br><br>DOI not provided	Partial response, for at least 5 years, to three different types of APs (at least two with different chemical structures); intolerance to adverse effects; and relapses or symptomatic deterioration despite the use of appropriate doses of the drugs	---	DRD2 141 Ins C allele homozygous genotype (Low high-density lipoprotein in TRap)
Rajagopal et al. (2018)<br><br>India<br><br>Pharmacogenet Genomics	n = 59 (treatment-responsive)<br>n = 34 (TRap)<br><br>35.3 ± 9.4 years (treatment-responsive)<br>34.8 ± 9.5 years (TRap)<br><br>75.0% male (treatment-responsive)<br>68.0% male (TRap)<br><br>12.7 ± 7.5 years (DOI for treatment-responsive)<br>11.4 ± 5.4 years (DOI for TRap)	At least 3 periods of treatment in the preceding 5 years with neuroleptic agents (from at least 2 different chemical classes) at dosages equivalent to or greater than 1000mg/d of chlorpromazine for a period of 6 weeks, each without significant symptomatic relief, and no period of good functioning within the preceding 5 years	---	COMT Val/Met or Met/Met + DRD4 120/120 or 120/240 (better clinical response to CLZ vs. those without these alleles)<br><br>COMT Val/Val + DRD4 120/120 or 120/240 (poorer response to CLZ)
Sagud et al. (2018)<br><br>Croatia<br><br>Front Pharmacol	n = 270 (SCZ with TRap)<br>n = 661 (SCZ non-TRap)<br><br>Median (25, 75 percentile):<br>39.0 (30,45) years (male Trap)<br>48.0 (38,58) years (female TRap)	Failure of at least two APs, given at ≥600 mg chlorpromazine equivalents (Inada and Inagaki, 2015) for more than consecutive 6 weeks, assessed retrospectively		COMT rs4680 AA genotype carriers (females with TRap)<br>COMT rs4818 CC genotype carriers (females with TRap)<br>rs4818-rs4680 non G-G/G-G haplotypes (females with TRap)

	40.0 (31,49) years (male non-TRap)<br>50.0 (39,57) years (female non-TRap)<br><br>79.6% male (SCZ with TRap)<br>49.9% male (SCZ non-TRap)<br><br>DOI not provided			
Akamine et al. (2017)<br><br>Japan<br><br>Ann Clin Biochem	n = 45 (SCZ on CLZ)<br><br>36.4 years<br><br>24.4% male<br><br>DOI not provided	All patients had received CLZ for at least four weeks (did not mention TRS criteria)		ABCG2 421 A allele carriers (Higher median C0/D of CLZ vs. those with the C/C genotype)
Kinoshita et al. (2017)<br><br>Japan<br><br>Int J Mol Sci	n = 21 (SCZ on CLZ)<br><br>42.1 ± 11.4 years<br><br>38.1% males<br><br>DOI not provided	(1) Non- or little response to treatment from at least two adequately dosed AP trials for at least 4 weeks (including at least one second-generation AP, >600 mg/day of chlorpromazine equivalent) and patients never had GAF scores that were higher than 40<br>(2) Intolerance to at least two second-generation APs because of uncontrolled extrapyramidal symptoms	---	Increased DNA methylation in a CpG site associated with CREBBP (CREB binding protein) cg05151055 (greater reduction in PANSS scores after CLZ treatment)
Mostaid et al. (2017)<br><br>Australia<br><br>Front Psychiatry	n = 71 (TRap)<br>n = 57 (HC)<br><br>40.0 ± 10.0 years (TRap)<br>40.0 ± 11.0 years (HC)<br><br>75.0% male (TRap)<br>61.0% male (HC)<br><br>17.0 ± 8.0 years (DOI for TRap)	Failed to respond to two or more previous trials of APs, had poor functioning, and persistent symptoms	---	P70S6K expression (elevated in TRap vs. HC)
Pinheiro et al. (2017)<br><br>Brazil	n = 54 (TRap)<br>n = 78 (HC)	Non-response to two different AP trials for at least 6 weeks. All patients selected were being treated with CLZ for at least one	---	GSTT1-null/GSTM1-null double-null genotype (increased risk for developing TRap)

PLoS One	38.7 ± 9.9 years (TRap)<br>39.0 ± 8.1 years (HC)<br><br>64.8% male (TRap)<br>61.5% male (HC)<br><br>DOI not provided	year to ensure the diagnosis of TRap		
Alacam et al. (2016)<br><br>Turkey<br><br>Psychiatr Res	n = 18 (TRap)<br>n = 19 (Treatment responsive)<br>n = 10 (HC)<br><br>41.6 ± 9.6 years (TRap)<br>39.1 ± 12.1 years (Treatment responsive)<br>31.5 ± 8.0 years (HC)<br><br>77.8% male (TRap)<br>68.4% male (Treatment responsive)<br>60.0% male (HC)<br><br>21.1 ± 7.5 years (DOI for TRap)<br>7.3 ± 8.2 years (DOI for treatment responsive)	1. Historical: at least three drug trials of different chemical classes with doses equivalent to 1000 mg/day chlorpromazine for a period of 6 weeks, without significant relief.<br>2. No period of good function in the preceding 5 years.<br>3. Actual:<br>a. A score of at least 45 in the BPRS, with scores of at least 4 in two of the following items: conceptual disorganization, suspiciousness, hallucinatory behavior, or unusual thought content.<br>b. CGI-Severity more than or equal to 4 (moderately ill).<br>4. Prospective: No improvement after 6 weeks of treatment with HAL (up to 60 mg/d or higher); improvement is defined as a 20% reduction of the BPRS as compared with the level of severity defined by the actual criteria and/or a post treatment CGI of ≤3 or a BPRS ≤35		Increased miR-181b-5p, miR-195-5p and miR-301a-3p expressions (in TRap vs. treatment-responsive SCZ)
Huang et al. (2016a)<br><br>USA<br><br>Pharmacogenomics	n = 208, separated by site of collection<br><br>n = 86 (57.0% TRap) (Cleveland site)<br>n = 90 (38.9% TRap) (Glen Oaks site)<br>n = 32 (62.5% TRap) (Irvine site)<br><br>32.4 ± 8.4 years (Cleveland site)<br>34.9 ± 8.6 years (Glen Oaks site)	(1) At least 3 periods of treatment in the preceding 5 years with neuroleptic agents (from at least 2 different chemical classes) at dosages equivalent to or greater than 1000mg/d of chlorpromazine for a period of 6 weeks, each without significant symptomatic	Responders were defined as those experiencing a reduction >20% in overall BPRS score after 6 months of treatment, based	DRD2 rs2514218 minor allele A (greater reduction in BPRS scores after CLZ)

	36.0 ± 6.7 years (Irvine site)<br><br>72.1% male (Cleveland site)<br>73.3% male (Glen Oaks site)<br>87.5% male (Irvine site)<br><br>DOI not provided	relief, and (2) no period of good functioning within the preceding 5 years	on criteria proposed by Kane et al.	
Huang et al. (2016b)<br><br>Taiwan<br><br>Psychiatr Genet	n = 143 (SCZ on CLZ)<br><br>50.1 ± 9.8 years<br><br>60.8% male<br><br>DOI not provided	Had received CLZ therapy for at least 14 days on a stable dose regimen		CYP1A2 163 A/A (lower CLZ plasma levels in SCZ on CLZ who are smokers)
Ruderfer et al. (2016)<br><br>Sweden<br><br>Lancet Psychiatry	n = 2536 (SCZ; n = 531 on CLZ)<br>n = 2543 (HC)<br><br>Age not provided<br><br>Gender not provided<br><br>DOI not provided	More than one AP trial at a particular dose and duration without improvement in symptoms; generally, two failed trials, with doses of 400–1000 chlorpromazine equivalent mg daily, for a duration of 4–6 weeks, with less than 20% improvement in the PANSS	---	Patients with TRap had an excess of rare disruptive variants in gene targets of antipsychotics (347 genes, p=0.0067) and in genes with evidence for a role in antipsychotic efficacy (91 genes, p=0.0029).
Terzić et al. (2016)<br><br>Slovenia<br><br>Psychiatr Danub	n = 94 (Treatment responsive)<br>n = 44 (TRap)<br>n = 94 (HC)<br><br>44.6 ± 10.8 years (Treatment responsive)<br>42.3 ± 11.2 years (TRap)<br>Age not provided (HC)<br><br>49.3% male (Treatment responsive + TRap)<br><br>15.5 ± 6.6 years (DOI for treatment responsive)<br>18.0 ± 7.9 years (DOI for TRap)	1. Drug-refractory condition: At least two prior drug trials of 4- to 6-weeks duration at 400 to 600 mg of chlorpromazine (or equivalent) with no clinical improvement<br>2. Persistence of illness: >5 years with no period of good social or occupational functioning<br>3. Persistent psychotic symptoms: BPRS total score >45 (on 18-item scale) and item score >4 (moderate) on at least two of four positive symptom items	---	---

Butcher et al. (2015)<br><br>Canada<br><br>Br J Psychiatry	n = 20 (22q11.2DS group)<br>n = 20 (Idiopathic group)<br><br>Median (25,75 percentile):<br>40.5 (23,53) years (22q11.2DS group)<br>46.0 (22,58) years (Idiopathic group)<br><br>55.0% male (22q11.2DS group)<br>85.0% male (Idiopathic group)<br><br>DOI not provided	Patients treated with CLZ	CGI-Improvement Scale	22q11.2DS-SCZ (respond as well to CLZ treatment as those with other forms of SCZ)
Chen et al. (2015)<br><br>Taiwan<br><br>J Clin Psychopharmacol	n = 55 (28 in metformin group, 27 in placebo)<br><br>Age for all patients not provided<br><br>Gender not provided<br><br>DOI not provided	Had taken CLZ for at least 3 months		SH2B1 minor allele carriers (higher blood pressure vs. SH2B1 common homozygous group)<br><br>TMEM18 minor allele carriers (CT + TT genotypes) (a greater reduction in insulin levels)<br>TMEM18 and GNPDA2 minor allele carriers (more weight loss)
Klemettilä et al. (2015)<br><br>Finland<br><br>Eur Psychiatry	n = 190 (SCZ on CLZ)<br><br>Median (25,75 percentile):<br>42.9 (20,67) years<br><br>57.4% male<br><br>DOI not provided	On CLZ		Leptin levels (positive correlation with BMI, levels of adipsin and IL-1Ra in all patients)<br>Leptin levels (positive correlation with IL-6 and weight gain in female patients only)<br>Leptin levels (positive correlation with level of triglycerides in male patients only)<br><br>Adipsin levels (positive correlation with IL-1Ra and IL-6 in all patients)<br>Adipsin levels (positive correlation with BMI in female patients only)
Terzić et al. (2015a)<br><br>Slovenia<br><br>J Mol Neurosci	n = 94 (Treatment responsive)<br>n = 44 (TRap)<br><br>44.6 ± 10.8 years (Treatment	Patients in this group had not responded to treatment with at least two different APs (at least one atypical) at doses equivalent to more than 400 to 600 mg		---

	responsive)<br>42.3 ± 11.2 years (TRap)<br><br>42.6% male (Treatment responsive)<br>63.6% male (TRap)<br><br>DOI not provided	chlorpromazine, taken for at least 6 weeks		
Terzić et al. (2015b)<br><br>Slovenia<br><br>Neuropsychiatr Dis Treat	n = 138 (SCZ group: 94 treatment responsive, 44 TRap)<br>n = 94 (HC)<br><br>Age not provided for both groups<br><br>63.6% male (TRap)<br>42.6% male (Treatment responsive)<br>Gender not provided (HC)<br><br>DOI not provided	Patients who did not respond to treatment with at least two different APs (at least one being atypical) at doses equivalent to more than 400–600 mg chlorpromazine, for a period of 6 weeks		---
Yang et al. (2015)<br><br>China<br><br>Prog Neuropsychopharmacol Biol Psychiatry	n = 260 (MetS or metabolic syndrome group)<br>n = 361 (Non-Mets)<br><br>44.4 ± 10.8 years (MetS)<br>44.2 ± 11.0 years (Non-MetS)<br><br>39.6% male (MetS)<br>58.7% male (Non-MetS)<br><br>DOI not provided	Received monotherapy with CLZ for at least one year		A-allele of SREBF2 gene rs2267443 or rs1052717 (increased risk of metabolic syndrome)
Bilic et al. (2014)<br><br>Croatia<br><br>Gene	n = 92 (TRap)<br>n = 81 (Non-TRap)<br><br>Median (25,75 percentile):<br>44.0 (36.3,50.0) years (TRap)<br>38.0 (31.3,49.0) years (Non-TRap)<br><br>46.7% male (TRap)	1) At least five years of inadequate social or occupational functioning, as determined by information gathered from medical documentation (placement in nursing care home or foster family, early retirement because of psychosocial disability brought on by SCZ)		SERT-PR SS genotype (development of TRS)

	50.6% male (Non-TRap)<br><br>Median (25,75 percentile):<br>14.0 (9.0,21.8) years (DOI for TRap)<br>9.0 (5.5,17.5) years (DOI for non-TRap)	2) Current treatment with APs medications in doses equivalent to, or higher than: 600 mg of chlorpromazine; PANSS score indicating moderate to severe psychopathology (score of 3 or higher) on following items: conceptual disorganization, suspiciousness, delusions and hallucinations; CGI-Severity score of at least 4 (moderate).<br>3) History of previous treatment with at least two APs from different groups using chlorpromazine equivalent doses of at least 600 mg; history of at least one treatment with CLZ using doses of at least 300 mg daily.<br>4) At 30 day-evaluation, TR patients still present with moderate to severe psychopathology in PANSS scale and are rated with the score of at least 3 on the following items: conceptual disorganization, suspiciousness, delusions and hallucinations; also, at the time of follow-up evaluation those patients had to have the CGI-Improvement score of 4 (no change) or higher (worse). TR patients were treated as inpatients (because of compliance concerns) for at least a month after being enrolled in the study, and any medication use during that period has been monitored		
Kang et al. (2014)<br><br>South Korea<br><br>Psychiatr Genet	n = 113 (SCZ on CLZ)<br><br>39.5 ± 8.3 years<br><br>71.7% male	Medicated with CLZ for more than 1 year. CLZ almost always prescribed for patients who are refractory to at least three APs		LEP-2548 AA group (greater gain in BMI than the AG group)

	19.0 ± 7.4 years (DOI)			
Li & Meltzer (2014)<br><br>USA<br><br>Schizophr Res	GWAS sample:<br>n = 79 (TRap)<br>n = 95 (Non-TRap)<br><br>20.4 ± 0.75 years (TRap, age at onset)<br>22.6 ± 0.83 years (Non-TRap, age at onset)<br><br>67.1% male (TRap)<br>66.3% male (Non-TRap)<br><br>DOI not provided<br><br>Additional sample:<br>n = 70 (TRap)<br>n = 125 (Non-TRap)<br><br>19.7 ± 1.3 years (TRap, age at onset)<br>20.9 ± 0.76 years (Non-TRap, age at onset)<br><br>64.3% male (TRap)<br>63.2% male (Non-TRap)<br><br>DOI not provided	Persistence of moderate to severe positive symptoms despite two or more trials of 4–6 week duration with typical or atypical APs other than CLZ		GRB10 gene rs2237457 (associated with TRap)
Solismaa et al. (2014)<br><br>Finland<br><br>Hum Psychopharmacol	n = 237 (SCZ on CLZ)<br>n = 388 (HC)<br><br>42.5 ± 11.0 years (SCZ on CLZ)<br>Age not provided for HC<br><br>57.4% male (SCZ on CLZ)<br>Gender not provided for HC<br><br>DOI not provided	On CLZ		ADRA2A (rs1800544) C/C genotype (associated with CLZ-induced sialorrhea vs. G-allele carriers)
Rajkumar et al. (2013)<br><br>India	n = 36 (CLZ non-responders)<br>n = 65 (CLZ responders)	Failure to respond at least two adequate AP trials, as documented by treating psychiatrists. An	BPRS total score of 35 or less	—

Acta Neuropsychiatr	35.4 ± 10.2 years (CLZ non-responders)<br>35.5 ± 9.1 years (CLZ responders)<br><br>63.9% male (CLZ non-responders)<br>76.9% male (CLZ responders)<br><br>11.1 ± 5.5 years (DOI for CLZ non-responders)<br>13.1 ± 7.3 years (DOI for CLZ responders)	adequate AP trial was defined by 600 mg chlorpromazine equivalents for a duration of at least 6 weeks with good drug compliance. The two adequate AP trials included at least one adequate trial with a serotonin dopamine antagonist.		
Zhang et al. (2013)<br><br>USA<br><br>Schizophr Res	n = 89 (SCZ on CLZ)<br>n = 190 (SCZ not on CLZ)<br><br>37.6 ± 10.0 years (SCZ on CLZ)<br>39.3 ± 10.5 years (SCZ not on CLZ)<br><br>70.8% male (SCZ on CLZ)<br>62.4% male (SCZ not on CLZ)<br><br>DOI not provided	Clinically assigned CLZ therapy was a proxy for treatment-resistance. All CLZ patients had failed at least 2 APs prior to initiating CLZ therapy	—	BDNF rs10767665, rs11030104, rs6265, rs10501087, rs11030096, rs4923460, rs6416056, rs10742178, rs4922788, rs1114029 (TRap)<br>Carrying minor allele at significant BDNF loci (increased risk of TRap)
Hwang et al. (2012)<br><br>USA<br><br>Prog Neuropsychopharmacol Biol Psychiatry	Sample characteristics for categorical measure of CLZ response (yes/no):<br>n = 183 (SCZ TRap Caucasians on CLZ, of which 53% were CLZ responders)<br>n = 49 (SCZ TRap African Americans on CLZ, of which 51% were CLZ responders)<br><br>34.4 ± 8.2 years (Caucasians)<br>34.9 ± 10.2 years (African Americans)<br><br>76.0% male (Caucasians)<br>69.0% male (African Americans)<br><br>DOI not provided<br><br>Sample characteristics for quantitative measure of CLZ response (using BRPS, BPOS, and BNEG):<br>n = 101 (SCZ TRap Caucasians on CLZ)	—	Responders to CLZ were defined as a reduction of more than 20% on the overall BPRS score after 6 months of treatment	DRD4 120-bp tandem repeat polymorphism 1-copy allele (CLZ non-responder status in African Americans)<br>DRD4 (G)n 142-bp/140-bp genotype group (poor CLZ response on BPRS and BPOS in combined sample)<br>DRD4 48-bp repeat polymorphism 4R allele (better CLZ response on BPRS and BPOS in Caucasians)<br>DRD4 48-bp repeat polymorphism short alleles ≤6R (better CLZ response on BPOS in Caucasians)<br>(G)n 142-bp allele-rs11246226 A allele haplotype (poorer response on BPRS in Caucasians)<br>(G)n 142-bp allele-rs936465 C allele haplotype (poorer response on BPRS in Caucasians)<br>rs11246226 A allele-rs936465 C allele haplotype (poorer response on BPOS in African Americans)

	n = 31 (SCZ TRap African Americans on CLZ)<br><br>33.8 ± 10.2 years (Caucasians)<br>34.9 ± 10.9 years (African Americans)<br><br>78.0% male (Caucasians)<br>68.0% male (African Americans)<br><br>DOI not provided			DRD5 rs10033951 T allele-rs100011006 G allele haplotype (better CLZ response on BNEG in Caucasians)
Rajkumar et al. (2012)<br><br>India<br><br>Psychopharmacology (Berl)	n = 101 (SCZ TRap on CLZ)<br><br>35.4 ± 9.4 years<br><br>72.3% male<br><br>12.4 ± 6.8 years (DOI for SCZ TRap on CLZ)	Failure to respond at least 2 adequate AP trials. An adequate AP trial was defined by 600 mg chlorpromazine equivalents for duration of at least 6 weeks with good drug compliance. Two AP trials included at least one adequate trial with a serotonin dopamine antagonist.	(1) Widely employed cross-sectional threshold of having BPRS total score of 35 or less; (2) BPRS total score below 25th percentile; (3) BPRS total score below median value; (4) BPRS total score below 75th percentile; (5) all five proposed remission criteria BPRS items, suspiciousness, hallucinatory behaviours, grandiosity, conceptual disorganization, and unusual thought content, rated mild or less; and (6) not more than one of these five BPRS items, rated moderate or above	HTR3A minor alleles of rs1062613 (T allele) and rs2276302 (G allele) (good responses on BPRS to CLZ)<br>Past history of catatonia, smoking, cognitive dysfunction, and hypersomnolence combined with HTR3A pharmacogenetic association model could explain 38.0% of variability among CLZ response<br>rs1062613 (CLZ response as defined by BPRS total scores ≤35)<br>rs2276302 (CLZ response as defined by BPRS total score ≤38 and not more than 1 of the 5 proposed remission criteria by Andreasen et al. 2005)
Teo et al. (2012)	n = 240 (SCZ, of which 35.4% were TRap)	Little or no symptomatic response to multiple (≥2) AP trials each of an	—	—

Canada<br><br>Pharmacogenet Genomics	36.4 ± 10.4 years (All SCZ)<br><br>71.3% male (All SCZ)<br><br>DOI not provided	adequate duration ≥6 weeks and with an adequate dose (therapeutic range)		
Bishop et al. (2011)<br><br>USA<br><br>Hum Psychopharmacol	n = 95 (SCZ TRap)<br><br>37.9 ± 10.2 years<br><br>64.0% male<br><br>DOI not provided	Persistent psychotic symptoms for at least 2 years despite adequate separate trials with 3 AP drugs from two biochemical classes at doses ≥1000 chlorpromazine equivalents for 6 weeks or inadequate response to at least 2 prior AP agents	—	GRM3 rs1989796 and rs1476455 (presence of refractory global symptoms as measured by BPRS total scores)<br>rs1476455 CC genotype (higher BPRS scores/TRap vs. A-carriers)<br>rs1989795 TT genotype (higher BPRS scores/TRap vs. C-carriers)
Hotta et al. (2011)<br><br>Japan<br><br>Prog Neuropsychopharmacol Biol Psychiatry	n = 35 (SCZ TRap)<br>n = 92 (SCZ non-TRap)<br><br>32.6 ± 11.0 years (SCZ TRap)<br>33.9 ± 11.8 years (SCZ non-TRap)<br><br>40.0% male (SCZ TRap)<br>54.3% male (SCZ non-TRap)<br><br>10.7 ± 10.1 years (DOI for SCZ TRap)<br>9.4 ± 7.8 years (DOI for SCZ non-TRap)	(1) A sufficiently long treatment with at least two different APs and a sufficient daily dose that failed to satisfactorily provide improvement in symptoms (GAF was never higher than 41 points).<br>(2) At least one atypical AP must have also been used.<br>(3) The highest daily chlorpromazine equivalent dose of AP must have been ≥600 mg.<br>(4) The duration of medication with typical APs must have been more than 1 year, and the duration of atypical APs must have been at least 4 weeks.	—	—
Kang et al. (2011)<br><br>South Korea<br><br>Psychiatry Investig	n = 69 (SCZ on CLZ with metabolic syndrome)<br>n = 77 (SCZ on CLZ with no metabolic syndrome)<br><br>41.3 ± 5.6 years (with metabolic syndrome)<br>38.4 ± 8.2 years (no metabolic syndrome)<br><br>73.9% male (with metabolic syndrome)<br>68.8% male (no metabolic syndrome)	Did not state that patients were TRap but all were on CLZ	—	—

	19.0 ± 7.5 years (DOI for all SCZ)<br>19.8 ± 7.3 years (DOI for SCZ with metabolic syndrome)<br>18.3 ± 7.7 years (DOI for SCZ with no metabolic syndrome)			
Lett et al. (2011)<br><br>Canada<br><br>Schizophr Res	n = 169 (SCZ TRap on CLZ)<br><br>34.8 years<br><br>75.7% male<br><br>DOI not provided	—	Responders to CLZ were defined as a reduction of more than 20% on the overall BPRS score after 6 months of treatment	rs1045881C allele of NRXN1 (CLZ response)<br>rs1045881 C/C genotype (CLZ response under a dominant model)
Mouaffak et al. (2011a)<br><br>France<br><br>Pharmacogenomics J	n = 40 (Ultra TRS)<br>n = 99 (Treatment responsive SCZ)<br><br>35.9 ± 11.7 years (Ultra TRS)<br>31.9 ± 10.6 years (Treatment responsive)<br><br>62.5% male (Ultra TRS)<br>66.7% male (Treatment responsive)<br><br>DOI not provided	—	The ultra-TR phenotype is characterized as patients who experience no clinical, social and/or occupational remission in spite of treatment with CLZ and at least two periods of treatment with distinct conventional or atypical AP drugs<br><br>Responders = Experienced a partial or full remission within 6–8 weeks of continuous treatment with an atypical or a conventional AP drug (including CLZ). All patients who had received	Frequency of minor allele A of rs3738401 in DISC1 (higher in ultra-resistant SCZ vs. treatment responsive SCZ)<br>Presence of A-containing genotypes (AA + AG) of rs3738401 (greater frequency in ultra-resistant SCZ vs. treatment responsive SCZ)<br>Frequency of minor allele A of rs3738401 (over-representation in males with ultra-resistant SCZ)<br>Frequency of minor allele T of rs6675281 (higher in treatment responsive SCZ vs. ultra-resistant SCZ)

			more than one AP and responded to treatment at least once were considered to be responders. Remission criteria were total 18-item BPRS score of ≤30 with no more than one item scoring 4, a CGI score of <3 and a GAF score of ≥61	
Mouaffak et al. (2011b)<br><br>France<br><br>Pharmacogenomics	n = 28 (Ultra TRS)<br>n = 78 (Treatment responsive SCZ)<br><br>34.7 ± 9.9 years (Ultra TRS)<br>31.9 ± 11.3 years (Treatment responsive SCZ)<br><br>64.3% male (Ultra TRS)<br>64.1% male (Treatment responsive SCZ)<br><br>DOI not provided	—	The ultra-TR phenotype is characterized as patients who experience no clinical, social and/or occupational remission in spite of treatment with CLZ and at least two periods of treatment with distinct conventional or atypical AP drugs<br><br>Responders = Experienced a partial or full remission within 6–8 weeks of continuous treatment with an atypical or a conventional AP drug (including CLZ). All patients who had received	Allelic CCAC combination of UCP4 rs3757241, rs10807344, rs9395206, and rs2270450 (under-represented in ultra-TR group vs. treatment responsive)

			more than one AP and responded to treatment at least once were considered to be responders. Remission criteria were total 18-item BPRS score of ≤30 with no more than one item scoring 4, a CGI score of <3 and a GAF score of ≥61	
Kohlrausch et al. (2010)<br><br>Brazil<br><br>J Psychiatr Res	n = 64 (CLZ responders)<br>n = 52 (CLZ non-responders)<br><br>33.9 ± 8.0 years (CLZ responders)<br>33.7 ± 9.1 years (CLZ non-responders)<br><br>85.9% male (CLZ responders)<br>84.6% male (CLZ non-responders)<br><br>15.1 ± 8.3 years (DOI for all SCZ)	—	30% reduction of the scores of the BPRS for appropriate response with drug adjustment up to 900 mg/day	Frequency of HTTLPR/rs25531 S'-allele (lower-expressing allele) (higher in CLZ non-responders vs. responders)<br>HTTLPR/rs25531 S'/S' homozygous (lower-expressing genotype) or S'/L' heterozygous (intermediate-expressing genotype) (more likely found in CLZ non-responders)
Souza et al. (2010)<br><br>USA	n = 140 (SZ TRap on CLZ)<br><br>Age not provided	Persistent psychotic symptoms for at least two years despite adequate separate trials with three APs from two biochemical classes at doses	Treatment response was expressed as a dichotomous	GFRA2 1-1-2 SNP27-SNP34-SNP37 haplotype carriers (Increased likelihood of responding to CLZ)

J Psychiatr Res	68.6% males<br><br>DOI not provided	≥1000 chlorpromazine equivalents for 6 weeks	variable at 6 months: a reduction of ≥20% on the overall score of the BPRS from the baseline score at enrolment	
Xu et al. (2010)<br><br>China<br><br>Prog Neuropsychopharmacol Biol Psychiatry	n = 114 (SCZ CLZ responders)<br>n = 46 (SCZ CLZ non-responders)<br><br>39.8 ± 13.7 years (SCZ CLZ responders)<br>42.6 ± 12.5 years (SCZ CLZ non-responders)<br><br>52.6% male (SCZ CLZ responders)<br>52.2% male (SCZ CLZ non-responders)<br><br>DOI not provided	—	Responders were defined as showing a minimum 40% decrease in the BPRS general score after treatment	Frequency of SLC6A3 gene rs2975226 -71T allele (higher in CLZ responders vs. CLZ non-responders)<br>Frequency of SLC6A3 gene rs2975226 -71A allele (lower in CLZ responders vs. CLZ non-responders)<br><br>Haplotype block constructed from rs2652511 (T-844C), rs2975226 (T-71A), and rs2963238 (Intron 1: A1491C):<br>Frequency of haplotype T-T-A (Higher in CLZ responders than non-responders)<br>Frequency of haplotype C-A-C (Lower in CLZ responders than non-responders)
Zuo et al. (2009)<br><br>USA<br><br>Pharmacogenet Genomics	n = 85 (SCZ TRap on CLZ, of which 68.2% were European-Americans)<br><br>Age not provided<br><br>98.7% male<br><br>DOI not provided	Persisting psychotic symptoms despite adequate treatment trials of two or more APs at 1000mg chlorpromazine equivalents unless limited by adverse effects	R=(P1-P2)/(P1-B)×100%<br>where R = reduction rate of PANSS score<br>P1 = PANSS scores before treatment<br>P2 = PANSS scores after 3-month treatment<br>B = lowest possible score on the PANSS i.e. 16<br><br>Response to treatment was defined as R ≥20%	DTNBP1 Diplotype ACCCTC/GTTGCC (CLZ effect on PANSS total, positive, and general psychopathology scores all patients)<br>Diplotype ACCCTC/GCCGCC (CLZ effect on PANSS positive scores in European-American patients)<br>Haplotype ACCCTC x GCCGCC (CLZ effect on PANSS positive scores in European-American patients)<br>Haplotype ACCCTC x GTTGCC (CLZ effect on PANSS total, positive, and general psychopathology scores for all patients)<br><br>DTNBP1 Allele T of rs742105 (Better CLZ response in African-American patients vs. those with allele C)<br>Genotypes T/T + T/C of rs742105 (Better CLZ response in African-American patients vs. those with genotype C/C)

Ji et al. (2008a)<br><br>Japan<br><br>Neurosci Lett	n = 101 (SCZ TRap on CLZ)<br>n = 239 (SCZ non-TRap on CLZ)<br><br>50.0 ± 10.5 years (TRap)<br>56.0 ± 13.1 years (non-TRap)<br><br>66.3% male (TRap)<br>55.6% male (non-TRap)<br><br>33.6 ± 12.4 years (DOI for all SCZ)	Hospitalized for more than 1 year and had been receiving AP therapy at dosages of at least 1000 mg/day chlorpromazine equivalents for more than 1 year	—	rs1062613 of HTR3A T/T genotype (higher neuroleptic dosage during maintenance therapy)
Ji et al. (2008b)<br><br>Japan<br><br>Nagoya J Med Sci	n = 101 (SCZ TRap)<br>n = 244 (SCZ non-TRap)<br><br>50.1 ± 10.5 years (SCZ TRap)<br>56.2 ± 11.9 years (SCZ non-TRap)<br><br>66.3% male (SCZ TRap)<br>56.5% male (SCZ non-TRap)<br><br>33.6 ± 12.4 years (DOI for all SCZ)	Hospitalized for more than 1 year and had been receiving AP therapy at dosages of at least 1,000 mg/day chlorpromazine equivalents for more than 1 year	—	Genotype frequency of HTR3B -100_102delAAG polymorphism (different in TRap vs. non-TRap groups)<br>Frequency of minor allele -100_-102delAAG(del) (higher in TRap vs. non-TRap)
Kohlrausch et al. (2008)<br><br>Brazil<br><br>Pharmacogenomics	n = 121 (Combined group: SCZ TRap on CLZ)<br>n = 66 (subgroup: CLZ responders)<br>n = 55 (subgroup: CLZ non-responders)<br><br>34.0 ± 8.8 years (Combined group)<br>33.9 ± 7.9 years (CLZ responders)<br>34.1 ± 9.8 years (CLZ non-responders)<br><br>83.5% male (Combined)<br>84.8% male (CLZ responders)<br>81.8% male (CLZ non-responders)<br><br>15.2 ± 8.5 years (DOI for all SCZ TRap on CLZ)	Lack of satisfactory clinical response to at least 2 or more standard APs, administered at doses equivalent to at least 1000mg chlorpromazine, for a minimal period of 6 weeks, and poor functioning level over the past 5 years	30% reduction in scores on BPRS	Homozygosis for GNB3 T825 allele (More frequent among CLZ non-responders vs. responders)<br>Homozygosis for GNB3 C825 allele (More frequent among CLZ responders vs. non-responders)<br>Carriers of GNB3 T825 allele (higher risk of being CLZ non-responder)<br>Carriers of GNB3 T825 allele (higher risk of convulsive episode)
Souza et al. (2008)<br><br>USA<br><br>Psychopharmacology (Berl)	n = 140 (SZ TRap on CLZ)<br><br>Age not provided<br><br>68.6% males<br><br>DOI not provided	(1) At least 3 periods of treatment in the preceding 5 years with neuroleptic agents (from at least 2 different chemical classes) at dosages equivalent to or greater than 1000mg/d of chlorpromazine for a period of 6 weeks, each without significant symptomatic	Treatment response was expressed as a dichotomous variable at 6 months based on a reduction of ≥20% on the overall	—

		relief, and (2) no period of good functioning within the preceding 5 years	score of the BPRS from the baseline score at enrolment	
Dettling et al. (2007)<br><br>Germany<br><br>Pharmacogenomics J	n = 42 (SCZ on CLZ with CA)<br>n = 75 (SCZ on CLZ without CA)<br><br>45.3 ± 16.3 years (CA)<br>34.3 ± 10.8 years (without CA)<br><br>45.2% male (CA)<br>52.0% male (without CA)<br><br>DOI not provided	—	—	HLA-Cw7-B18 and Cw7-B39 (development of CA)<br>HLA-DRB5*0201-DRB4*000 (development of CA)<br>HLA-Cw7-B18-DRB5*000, Cw7-B39-DRB5*000, and Cw7-B44-DRB5*000 (development of CA)
Woodward et al. (2007)<br><br>USA<br><br>Schizophr Res	n = 86 (SCZ TRap on CLZ)<br><br>30.8 ± 8.1 years<br><br>73.3% male<br><br>10.0 ± 7.0 years (DOI)	—	—	COMT met/met and val/met (superior performance within the attention and verbal fluency domain at 6 month assessment after CLZ vs. val/val group)<br>COMT met/met and val/met (higher COWAT/verbal fluency scores at 6 week and 6 month assessments after CLZ vs. val/val groups)<br>COMT met/met and val/met (lower ACTT/working memory error scores at 6 week assessment after CLZ vs. baseline)
Zhang et al. (2007)<br><br>China<br><br>J Clin Psychopharmacol	n = 67 (Male SCZ on CLZ)<br>n = 35 (Female SCZ on CLZ)<br><br>47.1 ± 6.1 years (Male)<br>47.5 ± 6.7 years (Female)<br><br>65.7% male (All SCZ on CLZ)<br><br>21.9 ± 7.5 years (DOI for all SCZ on CLZ)	Unsure if TRS but all on CLZ		Leptin G2548 A/A genotype (less BMI gain after CLZ treatment vs. G/G or G/A group)<br>Males with G2548 A/A leptin genotype (less BMI gain after CLZ treatment vs. those with G allele)
Zai et al. (2006)<br><br>USA<br><br>Psychopharmacology (Berl)	n = 247 (TRap; Combined sample A, B, C)<br>n = 96 (TRap on SCZ; Sample A)<br>n = 94 (Sample B)<br>n = 57 (Sample C)<br><br>35.2 ± 9.3 years (TRap; Combined sample)	(1) At least 3 periods of treatment in the preceding 5 years with neuroleptic agents (from at least 2 different chemical classes) at dosages equivalent to or greater than 1000mg/d of chlorpromazine for a period of 6 weeks, each without significant symptomatic	BPRS change scores	G-308A TNF-α- presence of A allele in samples A and B only (improvement on overall BPRS score after 3 and 6 months of CLZ treatment

	32.7 ± 8.9 years (TRap on SCZ; Sample A)<br>34.8 ± 8.4 years (Sample B)<br>40.0 ± 9.7 years (Sample C)<br><br>74.9% male (TRap; Combined sample)<br>71.9% male (TRap on SCZ; Sample A)<br>74.5% male (Sample B)<br>80.7% male (Sample C)<br><br>DOI not provided	relief, and (2) no period of good functioning within the preceding 5 years		
Hwang et al. (2006)<br><br>USA<br><br>Eur Neuropsychopharmacol	n = 97 (Caucasians with TRS)<br>n = 35 (African-Americans with TRS)<br><br>33.8 ± 10.2 years (Caucasians)<br>34.9 ± 10.9 years (African-Americans)<br><br>78.0% males (Caucasians)<br>68.0% males (African-Americans)<br><br>DOI not provided	(1) At least 3 periods of treatment in the preceding 5 years with neuroleptic agents (from at least 2 different chemical classes) at dosages equivalent to or greater than 1000mg/d of chlorpromazine for a period of 6 weeks, each without significant symptomatic relief, and (2) no period of good functioning within the preceding 5 years	Change scores on BPRS, BPOS, and BNEG	In African-Americans only:<br>DRD2 TaqIB B1 T allele (better response to CLZ based on BPRS and BPOS vs. C allele)<br>DRD2 rs1125394 A allele (allele dosage effect for improvement on BPRS and BPOS)<br>Haplotype 1-2 in SNP window 4-5 was associated with better response to CLZ for both overall BPRS and BPOS
Fehsel et al. (2005)<br><br>Germany<br><br>J Clin Psychopharmacol	n = 4 (SCZ on CLZ, with agranulocytosis)<br>n = 11 (HC)<br><br>Age not provided<br><br>Gender not provided<br><br>DOI not provided	—	—	Elevated expression levels of the proapoptotic genes p53, bax α, and bik in neutrophils (SCZ on CLZ with agranulocytosis vs. HC)
Goldberger et al. (2005)<br><br>France<br><br>Am J Med Genet B Neuropsychiatr Genet	n = 193 (SCZ, of which 76% responded to APs)<br><br>Age not provided<br><br>69.0% males (SCZ responders)<br>64.0% males (SCZ non-responders)<br><br>DOI not provided	TRap = No clinical remission despite several trials with different APs for a sufficient duration and still requiring permanent day care; May Dencker score 5-7<br><br>Treatment-responding patients = At least one partial clinical remission allowing their discharge from hospital; May Dencker score 1-4	—	Frequency of (CGG)10 allele in the 5' UTR of the reelin gene (greater in TRap vs. HC)<br><br>(CGG)10-containing genotypes (greater odds ratio for TRap vs. other genotypes)
Hwang et al. (2005)	n = 183 (Caucasians with TRS)<br>n = 49 (African-Americans with TRS)	(1) At least 3 periods of treatment in the preceding 5 years with	A reduction of ≥20% on the	In African-Americans only:<br>DRD2 TaqIB B2 allele (higher frequency

USA<br><br>Neuropsychopharmacol (Berl)	 34.4 ± 8.2 years (Caucasians)<br>34.9 ± 10.2 years (African-Americans)<br><br>76.0% males (Caucasians)<br>69.0% males (African-Americans)<br><br>DOI not provided	neuroleptic agents (from at least 2 different chemical classes) at dosages equivalent to or greater than 1000mg/d of chlorpromazine for a period of 6 weeks, each without significant symptomatic relief, and (2) no period of good functioning within the preceding 5 years	overall score of the BPRS from the baseline score taken at enrollment into the study	in CLZ responders)<br>DRD 2 allele 1 of rs1125394 (higher frequency in CLZ responders)<br>DRD2 TaqIA allele A2 (higher frequency in CLZ responders)<br>DRD2 TaqIA allele A1 (associated with being a CLZ responder)<br>Haplotype 1–2–2, containing all three protective alleles TaqIB, rs1125294, and TaqIA (more strongly associated with being a responder than any individual allele)<br><br>In Caucasians only:<br>Haplotype 2–1–1 of window 5–6–7 (associated with being a CLZ responder)<br>Haplotype 1–1–2 of window 6–7–8 (associated with being a CLZ responder)
Kootstra-Ros et al. (2005)<br><br>Netherlands<br><br>Ann Clin Biochem	n = 33 (Smokers - SCZ on CLZ)<br>n = 25 (Non-smokers - SCZ on CLZ)<br><br>Age not provided<br><br>Gender not provided<br><br>DOI not provided	—	CLZ serum concentration	—
Müller et al. (2005)<br><br>USA<br><br>Neurosci Lett	n = 59 (Obese SCZ TRap randomly assigned to treatment with CLZ/OLA/RIS/HAL)<br><br>40.1 ± 9.5 years<br><br>77.0% male<br><br>18.9 ± 7.2 years (DOI)	(1) Persistent positive symptoms (hallucinations, delusions, or marked thought disorder) after at least 6 contiguous weeks of treatment, presently or documented in the past, with one or more typical APs at doses ≥600 mg/day in chlorpromazine equivalents, (2) poor level of functioning over the past 2 years, defined by the lack of competitive employment or enrollment in an academic or vocational program and not having age-expected interpersonal relations with someone outside the biological family of origin with whom ongoing regular contacts	Change in PANSS scores from baseline after treatment with either RIS, OLZ, CLZ, or HAL	SNAP-25 *Mnl*I polymorphism, T/T genotype (greater reduction of PANSS score post-treatment vs. T/G and G/G)<br>SNAP-25 *Tai*I polymorphism, C/C genotype (greater reduction of PANSS score post-treatment vs. T/T and T/C)<br><br>SNAP-25 *Mnl*I polymorphism, T/T genotype (more weight gain vs. T/G and G/G genotypes)<br>SNAP-25 *Tai*I polymorphism, C/C genotype (more weight gain vs. T/C and T/T genotypes)

		were maintained, (3) baseline total score ≥60 on the PANSS		
Theisen et al. (2004)<br><br>Germany<br><br>Psychiatr Genet	n = 97 (SCZ German on CLZ)<br><br>22.1 ± 7.7 years<br><br>58.8% male<br><br>DOI not provided	—	—	—
Chiu et al. (2003)<br><br>Taiwan<br><br>Neuropsychobiology	n = 193 (SCZ TRap Chinese)<br><br>39.8 ± 7.9 years<br><br>Gender not provided<br><br>DOI not provided	Persistent severe psychotic symptoms for at least 6 months while the patient received adequate treatment with two different conventional APs	—	—
Hong et al. (2003)<br><br>Taiwan<br><br>Neurosci Lett	n = 93 (SCZ Chinese on CLZ, out of which 61.3% responded to CLZ)<br>n = 198 (HC Chinese)<br><br>36.8 ± 8.1 years (SCZ)<br>35.7 ± 9.1 years (HC)<br><br>65.6% male (SCZ)<br>62.1% male (HC)<br><br>DOI not provided	—	Responders were defined by a minimum 20% decrease in BPRS total score after CLZ treatment	Increased frequency of BDNF Val/Val genotype (in CLZ responders vs. HC)
Inada et al. (2003)<br><br>Japan<br><br>Am J Med Genet B Neuropsychiatr Genet	n = 100 (SCZ - both TRap and treatment responsive)<br><br>Age not provided<br><br>Gender not provided<br><br>DOI not provided	Hospitalized for more than 1 year and had been receiving AP therapy at dosages of at least 1,000 mg/day chlorpromazine equivalents for more than 1 year	—	—

Tsai et al. (2003)<br><br>Taiwan<br><br>Schizophr Res	n = 205 (SCZ, out of which 48.3% were given CLZ)<br>n = 75 (SCZ on CLZ with TNF-alpha -308G/G genotype)<br>n = 22 (SCZ on CLZ with TNF-alpha -308G/A genotype)<br>n = 2 (SCZ on CLZ with TNF-alpha -308A/A genotype)<br><br>37.2 ± 8.4 years (all SCZ)<br>37.6 ± 7.9 years (G/G)<br>33.9 ± 8.2 years (G/A)<br>36.5 ± 5.0 years (A/A)<br><br>48.8% male (all SCZ)<br>66.7% male (G/G)<br>63.6% male (G/A)<br>50.0% male (A/A)<br><br>DOI not provided	—	Percentage of BPRS total score reduction [(baseline - post-treatment) x 100/baseline]<br><br>Items of the BPRS were also grouped into positive, negative, and general clusters	—
Hong et al. (2001)<br><br>Taiwan<br><br>Psychiatr Genet	n = 25 (SCZ TRap on CLZ with GRIN2B 2664C/C genotype)<br>n = 49 (SCZ TRap on CLZ with GRIN2B 2664C/T genotype)<br>n = 26 (SCZ TRap on CLZ with GRIN2B 2664T/T genotype)<br><br>36.0 ± 1.5 years (2664C/C)<br>36.5 ± 1.1 years (2664C/T)<br>38.4 ± 1.9 years (2664T/T)<br><br>64.0% male (2664C/C)<br>65.3% male (2664C/T)<br>65.4% male (2664T/T)<br><br>DOI not provided	Lack of satisfactory clinical response to at least 2 or more standard APs, administered at doses equivalent to at least 1000mg chlorpromazine, for at least 6 weeks	Treatment response was evaluated in 2 ways: (1) ≥20% reduction in BPRS total score, or (2) percentage of BPRS score reduction [(visit - baseline) x 100/baseline]<br><br>Items of the BPRS were also grouped into positive, negative, and general clusters	GRIN2B 2664C/C genotype (higher mean CLZ dosage vs. C/T and T/T genotypes) - not corrected for multiple comparisons

Masellis et al. (2001)<br><br>USA<br><br>Schizophr Res	n = 185 (SCZ TRap on CLZ, out of which 52.4% were CLZ responders)<br><br>33.7 ± 8.9 years<br><br>71.4% male<br><br>DOI not provided	(1) at least 3 periods of treatment in the preceding 5 years with neuroleptic agents (from at least 2 different chemical classes) at dosages equivalent to or greater than 1000mg/d of chlorpromazine for a period of 6 weeks, each without significant symptomatic relief, and (2) no period of good functioning within the preceding 5 years	A reduction of ≥20% in the BPRS score from baseline score at enrollment into the study<br><br>In cases where a patient was very close to the operational criteria for response (≥15% but <20%) but were clinically much improved, a reduction of at least one category on the CGI scale was considered in order to augment the definition of response	—
Tsai et al. (2001)<br><br>Taiwan<br><br>Schizophr Res	n = 97 (SCZ TRap on CLZ)<br><br>36.9 ± 8.0 years<br><br>64.9% males<br><br>DOI not provided	Lack of satisfactory clinical response to at least 2 or more standard APs, administered at doses equivalent to at least 1000mg chlorpromazine, for at least 6 weeks	BPRS score changes (post-treatment minus baseline) was used in the analysis of clinical response to CLZ - items of the BPRS were grouped into positive, negative, and general clusters	—

Yu et al. (2001)<br><br>Taiwan<br><br>Neuropsychobiology	n = 16 (5HT2A C/C genotype in SCZ TRap on CLZ)<br>n = 46 (5HT2A C/T genotype in SCZ TRap on CLZ)<br>n = 37 (5HT2A T/T genotype in SCZ TRap on CLZ)<br><br>37.8 ± 7.2 years (C/C)<br>37.7 ± 8.4 years (C/T)<br>36.2 ± 8.1 years (T/T)<br><br>56.3% male (C/C)<br>65.2% male (C/T)<br>67.6% male (T/T)<br><br>DOI not provided	Lack of satisfactory clinical response to at least 2 classes of APs, administered at doses equivalent to at least 1000mg chlorpromazine, after treatment for at least 6 weeks	—	5HT2A receptor 102C/C genotype (Event Related Potential change - higher N100 amplitude/normalization after CLZ treatment)
Bolonna et al. (2000)<br><br>UK<br><br>Neurosci Lett	n = 289 (SCZ TRap on CLZ)<br><br>Age not provided<br><br>Gender not provided<br><br>DOI not provided	—	An improvement of 20 GAS points was considered the threshold for response to CLZ	—
Hong et al. (2000)<br><br>Taiwan<br><br>Neuropsychobiology	n = 81 (APOE e4 negative SCZ TRap Chinese on CLZ)<br>n = 14 (APOE e4 positive SCZ TRap Chinese on CLZ)<br><br>37.7 ± 0.9 years (APOE e4 negative)<br>31.5 ± 1.7 years (APOE e4 positive)<br><br>63.0% male (APOE e4 negative)<br>78.6% male (APOE e4 positive)<br><br>DOI not provided	Lack of satisfactory clinical response to at least 2 or more standard APs, administered at doses equivalent to at least 1000mg chlorpromazine for at least 6 weeks	BPRS score changes (post-treatment minus baseline) was used in the analysis of clinical response to CLZ - items of the BPRS were grouped into positive, negative, and general clusters	—
Tsai et al. (2000)<br><br>Taiwan<br><br>Schizophr Res	n = 90 (SCZ TRap Chinese on CLZ)<br><br>37.1 ± 0.9 years<br><br>65.6% male<br><br>DOI not provided	Lack of satisfactory clinical response to at least 2 or more standard APs, administered at doses equivalent to at least 1000mg chlorpromazine, for at least 6 weeks	BPRS score changes (post-treatment minus baseline) was used in the analysis of clinical response to CLZ -	—

			items of the BPRS were grouped into positive, negative, and general clusters	
Joober et al. (1999)<br><br>Canada<br><br>J Psychiatry Neurosci	n = 63 (SCZ TRap)<br>n = 39 (SCZ responders to APs)<br>n = 90 (HC)<br><br>38.6 ± 6.9 years (SCZ TRap)<br>41.8 ± 10.3 years (SCZ responders to APs)<br>44.6 ± 12.8 years (HC)<br><br>74.4% male (SCZ TRap)<br>71.7% male (SCZ responders to APs)<br>52.8% male (HC)<br><br>20.3 ± 7.0 years (DOI for SCZ TRap)<br>17.9 ± 8.4 years (DOI for SCZ responders to APs)	Continuous psychotic symptoms with no significant remission within the past 2 years, at least 3 periods of treatment with typical neuroleptics at optimal clinical requirements with no significant relief of symptoms in the preceding 5 years, and the inability to function without supervision in all or nearly all domains of social and vocational activities within the last 12 months	—	Higher frequency of genotype 2/2 of 5HT2A receptor gene (male TRap vs. male HCs)
Lin et al. (1999)<br><br>Taiwan<br><br>Neuroreport	n = 36 (SCZ TRap on CLZ with 102T/102T genotype)<br>n = 47 (SCZ TRap on CLZ with 102T/102C genotype)<br>n = 14 (SCZ TRap on CLZ with 102C/102C genotype)<br><br>36.1 ± 1.4 years (102T/102T)<br>38.3 ± 1.2 years (102T/102C)<br>38.8 ± 1.9 years (102C/102C)<br><br>63.9% male (102T/102T)<br>66.0% male (102T/102C)<br>57.1% male (102C/102C)<br><br>DOI not provided	Lack of satisfactory clinical response to at least 2 classes of APs, administered at doses equivalent to at least 1000mg chlorpromazine, after treatment for at least 6 weeks	BPRS score changes (visit minus baseline) were used in the analysis of response to CLZ	—
Scharfetter et al. (1999)<br><br>Pakistan<br><br>Eur Neuropsychopharmacol	n = 32 (SCZ TRap on CLZ, out of which 65.6% responded to CLZ)<br><br>33.3 ± 9.3 years<br><br>75.0% male	—	Response was defined as BPRS reduction of at least 50% after 6 months of treatment	DRD3 Ser-9-Gly polymorphism, Gly-9 allele (higher odds for CLZ response vs. Ser-9 allele)<br>Higher frequency of Ser-9/Ser-9 (in CLZ non-responders)<br>Higher frequency of Ser-9/Gly-9 (in CLZ

	5 years on average (DOI for all SCZ TRap)			responders)<br>Gly-9/Gly-9 (found exclusively in CLZ responders)
Amar et al. (1998)<br><br>Israel<br><br>Int J Neuropsychopharmacol	n = 3 SCZ TRap on CLZ developed granulocytopenia (1 male Ashkenazi, 2 females non-Ashkenazis)<br>n = 2 SCZ TRap on CLZ developed agranulocytosis (1 male and 1 female, both non-Ashkenazis)<br>n = 7 SCZ TRap on CLZ did not develop granulocytopenia/agranulocytosis (7 non-Ashkenazis, 6 Ashkenazis)<br><br>Age not provided (for all groups)<br><br>Gender not provided (for all groups)<br><br>DOI not provided	Continuously ill for more than 5 years and had not responded to at least 2 trials of classical APs administered at adequate dosage and for the prescribed duration	—	Increased frequency of HLA-DQw2 allelic variant (SCZ CLZ who developed granulocytopenia/agranulocytosis)<br>Increased frequency of HLA-DQB1*0201 allelic variant (SCZ CLZ who developed agranulocytosis/granulocytopenia)
Malhotra et al. (1998)<br><br>USA<br><br>Mol Psychiatry	n = 68 (SCZ on CLZ)<br><br>38.4 ± 7.8 years<br><br>73.5% male<br><br>DOI not provided	—	Responders: At least 20% reduction in BPRS total scores after 10 weeks of CLZ treatment	—
Masellis et al. (1998)<br><br>USA<br><br>Neuropsychopharmacology	n = 185 (SCZ TRap on CLZ, out of which 52.4% responded to CLZ)<br><br>33.7 ± 8.9 years<br><br>71.4% male<br><br>DOI not provided	(1) at least 3 periods of treatment in the preceding 5 years with neuroleptic agents (from at least 2 different chemical classes) at dosages equivalent to or greater than 1000mg/d of chlorpromazine for a period of 6 weeks, each without significant symptomatic relief, and (2) no period of good functioning within the preceding 5 years	A reduction of ≥20% in the BPRS score from baseline score at enrollment into the study<br><br>In cases where a patient was very close to the operational criteria for response (≥15% but <20%) but were clinically much improved, a reduction of at least one category	Higher frequency of tyr452 in HTR2A (in CLZ non-responders)

			on the CGI scale was considered in order to augment the definition of response	
Valevski et al. (1998)<br><br>Israel<br><br>Eur J Immunogenet	n = 11 (SCZ Ashkenazi - developed agranulocytosis on CLZ)<br>n = 50 (SCZ Ashkenazi - did not develop agranulocytosis on CLZ)<br><br>43.4 ± 10.9 years (SCZ Ashkenazi - agranulocytosis)<br>Age not provided (SCZ Ashkenazi - no agranulocytosis)<br><br>36.4% male (SCZ Ashkenazi - agranulocytosis)<br>Gender not provided (SCZ Ashkenazi - no agranulocytosis)<br><br>DOI not provided	—	—	Higher frequency of HLA-B38 in SCZ Ashkenazi (agranulocytosis vs. HC)
Kohn et al. (1997)<br><br>Israel<br><br>Eur Neuropsychopharmacol	n = 98 (49 SCZ Ashkenazi, 49 SCZ non-Ashkenazi)<br><br>22.4% CLZ non-responders (SCZ Ashkenazi)<br>53.1% CLZ responders (SCZ Ashkenazi)<br><br>34.7% CLZ non-responders (SCZ non-Ashkenazi)<br>40.8% CLZ responders (SCZ non-Ashkenazi)<br><br>Age not provided (for all groups)<br><br>Gender not provided (for all groups)<br><br>DOI not provided	—	Definite responders: Marked symptomatic improvement and definite improvement in hospitalization status and functioning<br><br>Minimal responders: Partial symptomatic improvement, or definite symptomatic improvement but no accompanying change in hospitalization status and	—

			functioning<br><br>Non-responders: Symptomatic status unchanged or worse	
Shaikh et al. (1996)<br><br>UK<br><br>Hum Genet	n = 133 (SCZ TRap on CLZ - European, Caucasian origin)<br><br>Age not provided<br><br>Gender not provided<br><br>DOI not provided	All patients were either refractory to or intolerant of typical APs, or both (definition of TRap not provided)	Responders were defined as those showing a 20-point improvement on the pretreatment GAS ratings	Higher frequency of DRD3 (Ser-9-Gly) polymorphism, genotype 1-1 (CLZ non-responders)
Shaikh et al. (1995)<br><br>Europe (UK and France) and Taiwan<br><br>Am J Med Genet	n = 147 (SCZ TRap on CLZ - European, Caucasian origin)<br>n = 42 (SCZ TRap on CLZ - Taiwan, Chinese origin)<br><br>Age not provided (for both groups)<br><br>59.2% male (European, Caucasian origin)<br>47.6% male (Taiwan, Chinese origin)<br><br>DOI not provided	All patients received a minimum of 2 neuroleptics of different chemical classes at a dose of at least 600mg chlorpromazine equivalent for at least 1 month and failed to respond well	Post-treatment GAS score minus the pre-treatment GAS scores, so that the possible range is - 100 to 100	—
Lieberman et al. (1990)<br><br>USA<br><br>Arch Gen Psychiatry	n = 6 (SCZ TRap on CLZ - agranulocytosis)<br>n = 25 (SCZ TRap on CLZ- no agranulocytosis)<br><br>29.8 ± 6.1 years (agranulocytosis)<br>28.2 ± 4.9 years (no agranulocytosis)<br><br>67.0% male (for both patient groups)<br><br>DOI not provided	(1) at least 3 periods of treatment in the preceding 5 years with neuroleptic agents (from at least 2 different chemical classes) at dosages equivalent to or greater than 1000mg/d of chlorpromazine for a period of 6 weeks, each without significant symptomatic relief, and (2) no period of good functioning within the preceding 5 years	—	Increased frequency of HLA-B38, DR4, DQw3 haplotype (CA)

Abbreviations: AP = Antipsychotics; BMI = Body Mass Index; BNEG = Brief Psychotic Rating Scale Negative Symptoms; BPOS = Brief Psychotic Rating Scale Positive Symptoms; BPRS = Brief Psychotic Rating Scale; CA = Clozapine-induced agranulocytosis; CGI = Clinical Global Impression; CLZ = Clozapine; DOI = Duration of illness; GAF = Global Assessment of Functioning; GAS = Global Assessment Scale; GWAS = Genome Wide Association Study; HAL = Haloperidol; HC = Healthy control; OLA = Olanzapine; PANSS = Positive and Negative Syndrome Scale; SCZ = Schizophrenia; TRap = Treatment resistance to antipsychotics (not including Clozapine); TRS = Treatment-resistant Schizophrenia.
